# Symbolic Interactionism and Grounded Theory in research on the health of children and adolescents: a scoping review^1^

**DOI:** 10.1590/1518-8345.7900.4933

**Published:** 2026-10-09

**Authors:** Marimeire Morais da Conceição, Climene Laura de Camargo, Maria Ribeiro Lacerda, Maria Carolina Ortiz Whitaker, Amâncio António de Sousa Carvalho, Kamylla Santos da Cunha, Ariane Teixeira de Santana, Telmara Menezes Couto, Eliana Rosa da Fonseca, Cristiane Cardoso de Paula

**Affiliations:** 1 Universidade Federal da Bahia, Salvador, BA, Brazil.; 2 Secretaria de Saúde da Bahia, Escola de Saúde Pública da Bahia Professor Jorge Novis, Salvador, BA, Brazil.; 3 Scholarship holder at the Coordenação de Aperfeiçoamento de Pessoal de Nível Superior (CAPES), Brazil.; 4 Scholarship holder at the Conselho Nacional de Desenvolvimento Científico e Tecnológico (CNPq), Brazil.; 5 Universidade Federal do Paraná, Curitiba, PR, Brazil.; 6 Universidade de Trás-os-Montes e Alto-Douro, Escola Superior de Saúde, Quinta dos Prados, Vila Real, Portugal.; 7 Centro de Investigação em Estudos da Criança, Universidade do Minho, Braga, Portugal.; 8 Universidade Federal de Santa Catarina, Florianópolis, SC, Brazil.; 9 Governo Municipal, Secretaria Municipal de Saúde de Biguaçu, Biguaçu, SC, Brazil.; 10 Universidade Federal do Rio de Janeiro, Rio de Janeiro, RJ, Brazil.; 11 Universidade Federal da Santa Maria, Santa Maria, RS, Brazil.; 12 Instituto JBI, Centro Colaborador, São Paulo, SP, Brazil.

**Keywords:** Symbolic Interactionism, Grounded Theory, Adolescent Health, Child, Review, Health.

## Abstract

**Objective:**

to map the theoretical-methodological application of Symbolic Interactionism and Grounded Theory in research on the health of children and adolescents.

**Method:**

a scoping review conducted in accordance with the recommendations of the JBI. The following elements were considered: Participants - children and/or adolescents, family members, and professionals; Concepts - Symbolic Interactionism and Grounded Theory; Context - not delimited. Nine electronic sources were consulted, including original articles, without language or time restrictions. Rayyan and NVivo software were used for study selection and data synthesis.

**Results:**

fifty-nine studies were included. Most participants were adolescents (49%) and family members (40%). Regarding the theoretical perspective, 42% adopted Blumer’s framework and 67% Strauss’s methodology. The combination of data collection strategies was reported in 34% of the studies; analytical resources such as diagrams and memos were cited in 42% and 39% of the studies, respectively. Only 24% reported theory validation. The hospital setting was the most frequently investigated context (27%).

**Conclusion:**

the combination of data collection strategies enables greater analytical diversity and investigations across different sociocultural and intersectoral contexts, contributing to the development of substantive theories. Future studies including the simultaneous participation of children, adolescents, family members, and professionals are recommended.

## Highlights

**(1)** Adolescents and family members are the most frequently investigated social groups.

**(2)** Strauss’s works are the most frequently cited methodological references.

**(3)** The combination of data collection strategies is an underutilized resource.

**(4)** Analytical tools such as diagrams and memos are underutilized.

**(5)** Fewer than one quarter of the studies mention validation of the substantive theory.

## Introduction

Symbolic Interactionism (SI) is a sociological theory that emerged in the late eighteenth century and gained prominence in the 1930s. In this context, scholars sought to explain the relationship between individuals and society through social interactions, as well as the implications of these interactions for individuals’ psychological processes^1^ ^-^ ^2^. SI is based on three premises: 1) human beings act toward things/situations based on the meanings they attribute to them; 2) the meaning of these things/situations arises from social interaction; and 3) such meanings are modified through individuals’ interpretative processes when dealing with them. Within these premises, SI is grounded in six central concepts: society, symbol, mind, self, structure, and social interaction^2^.

Thus, SI introduces discussions concerning the social being, whose theoretical foundations are anchored in the ideas of George Herbert Mead and later organized and systematized by Herbert Blumer, who compiled, structured, and recorded Mead’s thoughts^1^ ^-^ ^2^. These ideas were presented in lectures, meetings, and seminars at the University of Chicago, where Anselm Strauss received his academic training^2^ ^-^ ^3^. 

Strauss was influenced by SI and, together with the sociologist Barney Glaser, developed Grounded Theory (GT)^3^. GT is a research methodology originally aligned with positivist thought. However, Strauss incorporated interpretivist analytical procedures that dialogue with SI^2^ ^-^ ^3^, giving rise to the theoretical foundations of GT within qualitative research^3^.

When employing GT, researchers aim to identify strategies developed by individuals in dealing with various social objects, particularly situations experienced within their life contexts and in interaction with the broader society to which they belong^3^ ^-^ ^5^. For this reason, GT is currently recognized for its effectiveness in studies seeking to generate formal and substantive theories^3^ ^-^ ^6^. In continuity with the original purpose of Glaser and Strauss, the method is suitable for addressing social themes and processes considered complex and multifaceted^3^ ^,^ ^7^, as is often the case in health research. 

In the present study, the term health is understood in accordance with the concept proposed by the World Health Organization (WHO), which adopts a multidimensional perspective. Thus, it encompasses not only biological, behavioral, and socioeconomic dimensions, but also environmental conditions and political relationships^8^ ^-^ ^9^. Scholars emphasize the central role of interactions in promoting actions aimed at the health of children and adolescents^10^ ^-^ ^13^. Therefore, it is relevant to focus on the theoretical-methodological application of GT in association with SI in scientific investigations addressing the health of this population, as both frameworks support the understanding of relationships established among individuals within society.

This social experience begins in utero^14^ ^-^ ^15^, is guided during childhood and adolescence by the family (primary socialization), and continues throughout life with the involvement of health and education professionals (secondary socialization), as demonstrated by studies conducted in Brazil, China, the Netherlands, and the United States of America (USA)^11^ ^-^ ^12^ ^,^ ^16^ ^-^ ^17^. In the current context of rights recognition, it has become increasingly common for children and adolescents to have access to information about themselves and their health conditions^18^ ^-^ ^21^, a scenario that enables participation in decision-making and self-care, in line with autonomy and youth protagonism^20^ ^,^ ^22^ ^-^ ^24^, elements that contrast with adult-centered care^19^ ^,^ ^21^ ^,^ ^25^. 

This study identifies scientific gaps that may support the development of new investigations addressing emerging themes, through the use of diverse data collection strategies and analytical resources aimed at theory development. It also provides a synthesis of the theoretical-methodological application of SI and GT in research, while encouraging the expansion of studies in innovative and previously unexplored sociocultural contexts. Finally, the objective of this review was to map the theoretical-methodological application of Symbolic Interactionism and Grounded Theory in research on the health of children and adolescents.

## Method

### Type of study 

This was a Scoping Review (ScR) conducted in accordance with the recommendations proposed by the JBI^26^ ^-^ ^27^ ^)^ and reported following the PRISMA Extension for Scoping Reviews (PRISMA-ScR)^28^. The protocol was registered in the Open Science Framework (OSF): https://doi.org/10.17605/OSF.IO/BKTMJ. 

The review team consisted of three professionals with theoretical and methodological expertise in SI and GT: one reviewer with expertise in child and adolescent health, one methodological consultant with expertise in JBI scoping reviews, and one librarian with expertise in review studies in the health field.

### Review question 

The PCC mnemonic^27^ was applied, representing: (P) Population - children, adolescents, family members, and/or professionals; (C) Concept - SI and GT; and (C) Context - any setting. Based on this framework, the guiding question was formulated as follows: how are SI and GT theoretically and methodologically applied in research on the health of children and adolescents? 

Secondary questions were established to guide the mapping process: Which social groups are addressed in studies on child and adolescent health? Which analytical and methodological tools are used in knowledge production? 

### PCC components

The following *participants* were considered: 1) children and adolescents aged two to under 18 years, according to the World Health Organization (WHO) framework^29^; 2) family caregivers: individuals who hold legitimate authority over the child and/or adolescent, have a family relationship and/or maintain kinship (or not), such as father/mother, stepfather/stepmother, grandparent, uncle/aunt, sibling, cousin^29^ ^-^ ^31^; 3) non-family caregivers: individuals who care for children and/or adolescents but are not family members or health professionals, such as babysitters, teachers, supervisors, and coaches^29^ ^,^ ^32^; 4) health professionals: individuals with training in the health field, such as nursing staff, physicians, nutritionists, physiotherapists, speech therapists, social workers, psychologists, occupational therapists, and Community Health Workers (CHWs)^29^ ^-^ ^30^ ^,^ ^33^; and 5) professionals without health training, such as educators and child protection and security agents^30^. 

Regarding the *concept*, only studies investigating the theoretical-methodological application of SI in research conducted using the GT method were included. SI posits that individuals respond to social objects (objects, speech, actions, people) by resorting to symbols and attributing meanings to them^1^ ^-^ ^2^.

GT is a scientific research methodology that guides theory construction through the grouping of concepts. To this end, it relies on simultaneous data collection and analysis, rigorously systematized to identify concepts and demonstrate how they relate to one another around a central phenomenon. The theory that emerges from the data explains individuals’ experiences and justifies their reactions to the events they encounter^3^ ^,^ ^7^. 

Any national or international context was considered eligible, including any setting related to child and adolescent health care. 

The inclusion criteria comprised: articles published in peer-reviewed journals, resulting from field research, original empirical studies of any approach, grounded in SI and conducted methodologically using GT. Due to the possibility of data duplication extracted from the methods of selected studies, gray literature was not included. Studies adopting more than one theoretical-methodological framework were excluded. 

### Data collection

The search strategy was developed by the librarian who was part of the review team. Based on the acronym, search terms were identified in controlled vocabularies - Health Sciences Descriptors (DeCS), Medical Subject Heading (MeSH) and Embase Subject Headings (Emtree) - and two preliminary searches were conducted. After defining the search strategy (Figure 1), it was adapted to each database.

**Figure 1:** Search strategy applied to MEDLINE/PubMed on February 8, 2023, updated on July 10, 2024

Search	Query	Results
#1	Search: “Grounded Theory”[mh] OR Grounded Theory[tiab] Sort by: Most Recent	14,791
#2	Search: “Symbolic Interactionism”[mh] OR Symbolic Interactionism*[tiab] OR symbolic interaction[tiab] OR symbolic interaction theory[tiab] OR “Social interaction”[mh] OR Social Functioning*[tiab] OR Social Interaction*[tiab] Sort by: Most Recent	40,810
#3	Search: “Infant”[mh] OR Infant*[tiab] OR “Child, Preschool”[mh] OR Preschool*[tiab] OR “Child”[mh] OR Child*[tiab] OR Children[tiab] OR Pediatric*[tiab] OR “Adolescent[mh]” OR Adolescence[tiab] OR Adolescent*[tiab] OR Teen*[tiab] OR Teenager*[tiab] OR Youth*[tiab] OR “Child Health”[mh] Sort by: Most Recent	3,519,518
#4	Search: (#1 AND #2) AND #3 Sort by: Most Recent	84

Searches were conducted in the following sources: MEDLINE (PubMed), Scientific Electronic Library Online (SciELO), Nursing Database (via the Virtual Health Library Portal), Cumulative Index to Nursing and Allied Health Literature (CINAHL), Education Resources Information Center (ERIC),  Academic Search Scopus (EBSCO), Embase (Elsevier), Web of Science (Clarivate Analytics), and PsycINFO.

Initially, the 1,357 retrieved references were imported into EndNote to identify and remove duplicates and subsequently exported to Rayyan, a free web-based program funded by the Qatar Foundation^34^. Study selection was conducted independently by two reviewers according to the inclusion criteria. The first screening involved title and abstract review; the second involved full-text reading. In cases of disagreement between reviewers, a third reviewer was consulted for decision-making^27^. 

A total of 1,947 references were screened after the selection of 51 studies, and eight additional texts were included. Nine full texts that were not available were requested from the authors via email; however, only one author provided the complete text, which was incorporated into the results.

Reviewer 1 created an Excel spreadsheet and a codebook containing all variables and their respective descriptions to standardize data extraction^35^. A pilot test^27^ allowed for adjustments to the spreadsheet. In stage 1, the following data were extracted: authors, objective, theme, descriptors, year of publication, and country where the study was conducted.

In stage 2, the following elements were extracted: 1) participants: sample; participation of children and/or adolescents (number, age, sex, race/ethnicity); participation of family members (number, age, sex, race/ethnicity, relationship to the child/adolescent); professionals (number, age, sex, race/ethnicity, field of expertise); 2) concepts: regarding SI, cited authors, cited works, and application of the six core concepts; regarding GT, authors, cited works, methodological approach, data collection technique/instrument, duration of interviews, data collection period, memos, diagrams, number of codes generated, number of sampling groups, software used, validation, and type of validation; and 3) context: setting of the investigation (health services, education, community, associations), with health services classified as hospital, specialized, or primary care.

For data extraction, the selected material was carefully read by two independent reviewers^27^ ^,^ ^35^ ^-^ ^36^, then grouped and synthesized in Microsoft Excel and subsequently analyzed using NVivo software, version 14. In Excel, the articles received a numerical identifier from 01 to 59 according to year and order of publication.

The presence of PCC variables was recorded in the spreadsheet in a dichotomous manner, with single-word responses (e.g., “yes” or “no”), which underwent basic quantitative analysis^35^, generating percentages relative to the total number of articles. The geographical distribution of published studies was generated using an automated Excel formula based on the frequency of studies conducted in different countries. The word cloud was generated through automatic grouping of descriptors/keywords in NVivo. 

A basic qualitative descriptive content analysis^35^ was conducted, in which excerpts were selected, labeled with the numerical identifier assigned to the source article, transformed into codes, and grouped according to themes derived from the six foundational SI concepts^1^ ^-^ ^2^. Subsequently, the frequency of these elements was calculated.

## Results

A total of 59 articles were selected, published between 1998 and 2024; 33 (55.93%) were published in national journals, and 17 (28.81%) were published within the last five years. The results are presented in Figure 2 through the Preferred Reporting Items for Systematic Reviews and Meta-Analyses (PRISMA) flow diagram^37^:

**Figure 2:**
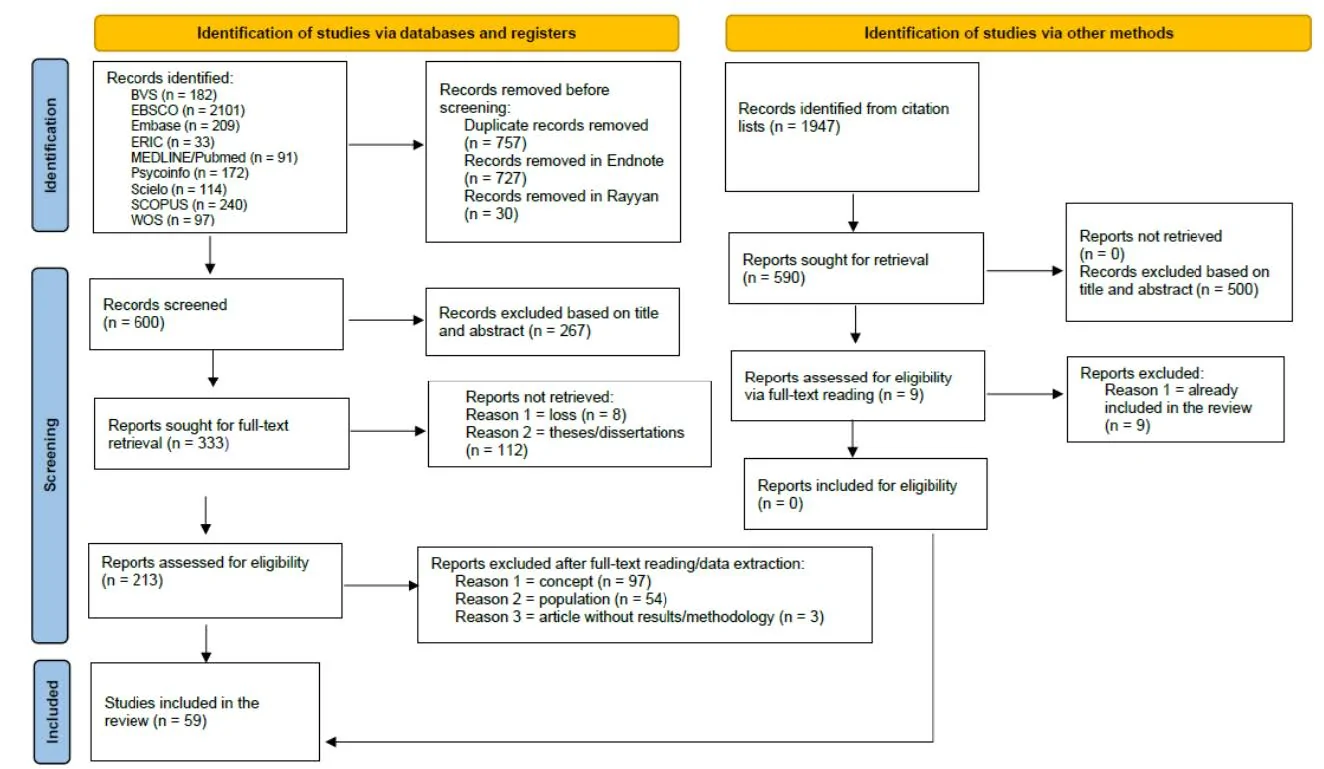
PRISMA flow diagram^37^ of study selection. Salvador, BA, Brazil, 2024

The studies were conducted across five continents: 35 (59.32%) in South America; 10 (16.94%) in Europe; 7 (11.86%) in North America; 2 (3.38%) in Asia; and 1 (1.69%) in Oceania. In South America, 31 (52.54%) were Brazilian studies, of which 29 (49.15%) were conducted in southeastern states, 1 (1.69%) in the southern region, and 2 (3.38%) did not report the region, state, or municipality where the research was conducted. The geographical distribution of the selected studies is shown in Figure 3:

**Figure 3:**
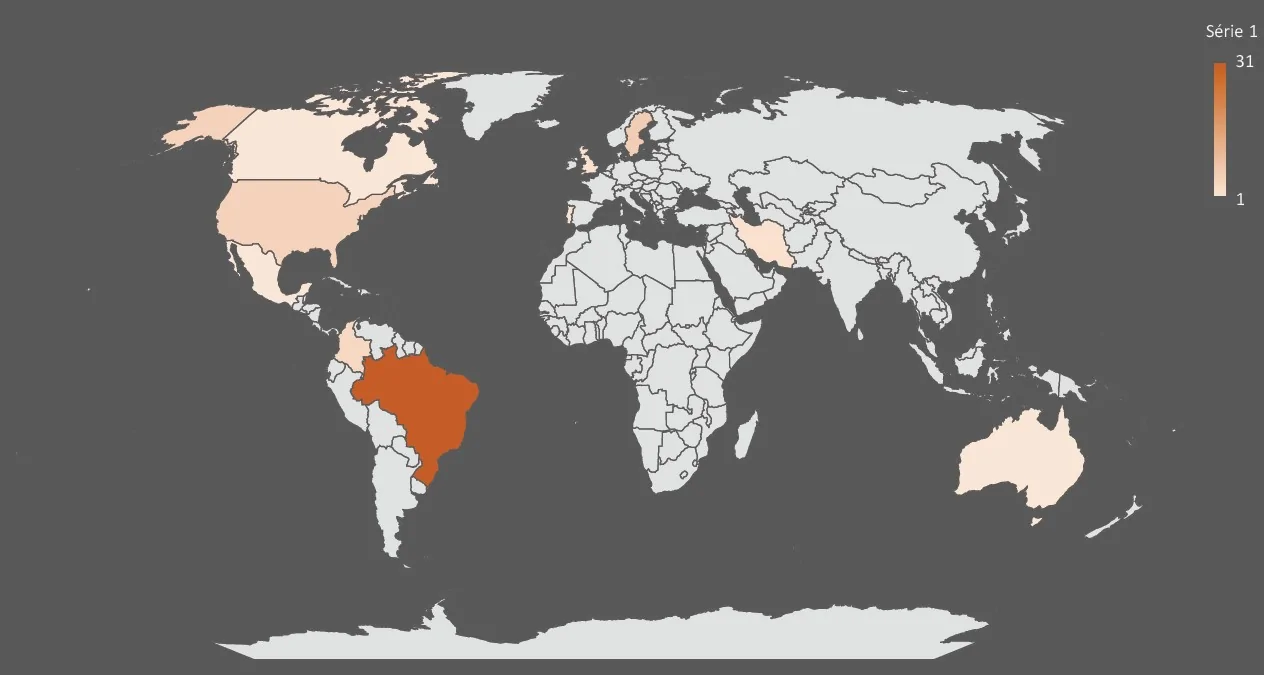
Geospatial distribution of studies reporting the research setting (n = 55). Salvador, BA, Brazil, 2024

Overall, 58 (98.30%) articles presented descriptors extracted from DeCS or MeSH. Among the descriptors used, the term “Nursing” (and related terms) appeared in 30 (50.84%) articles; the term “Child” appeared in 25 (42.37%) articles; and the expression “Grounded Theory” appeared in 11 (18.64%) articles, findings consistent with Figure 4:

**Figure 4:**
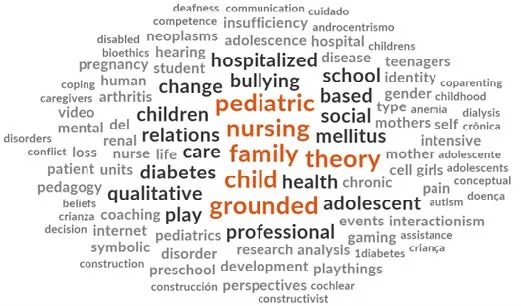
Word cloud generated from the descriptors/keywords of the articles (n = 58). Salvador, BA, Brazil, 2024

The authors investigated themes involving: diseases, 17 (28.81%); social relationships, 13 (22.03%) - related to interactions established among individuals^2^; and social processes, 9 (15.25%) - individual or collective responses to objects, situations, and social contexts^2^. Additionally, 7 (11.86%) studies addressed therapeutic interventions; 5 (8.47%) addressed health conditions; 3 (5.08%) focused on syndromes; 3 (5.08%) on disorders; and 2 (3.38%) on disabilities.

Figure 5 presents the studies in order from the most recent to the oldest publication.

**Figure 5:** Summary of the characteristics of the selected articles: author, country, objective, and sample. Salvador, BA, Brazil, 2024

Nº	Author	Objective	Sample
1	Juan-Martínez, et al.^(38)^	To understand reflections on the construction of gender identities and the implications of violence among Indigenous adolescents in northeastern Oaxaca.	45
2	Queiroz, et al.^(39)^	To analyze factors involved in the management of nursing care for hospitalized children with rheumatic heart disease.	19
3	Mazaherizadeh, et al.^(40)^	To explore underlying factors, processes, and consequences of gaming disorder among Iranian male adolescents and contextual factors related to gaming disorder within Iran’s unique sociocultural and psychological tapestry.	13
4	Picolo Gimenes, et al.^(41)^	To understand how nurses represent themselves experiencing the use of Therapeutic Play in the care of children and adolescents supported by Drawing-Story.	11
5	Rafii, et al.^(42)^	To explore the life process of mothers of children with autism.	14
6	Novoa-Villegas, et al.^(43)^	To analyze meanings attributed by boys and girls to the configuration of masculinity(ies) expressed in feminist children’s literature.	10
7	Giraldo-Giraldo, et al.^(44)^	To examine the relationship between the appropriation and use of social media and the construction of the self among adolescent students.	19
8	Moreira-Dias, et al.^(45)^	To present a theoretical model of the interactional context between health professionals and families of children and adolescents in palliative care.	10
9	Gimenes, et al.^(46)^	To understand how nurses interpret their role in interacting with children during the use of Therapeutic Play and to construct a theoretical model representing this experience.	11
10	Sethi^(47)^	To deepen existing knowledge about influences on fatherhood through an occupational lens.	12
11	Pérez-Rivera, et al.^(48)^	To present and discuss meanings and practices of fatherhood and masculinity in child-rearing that either reproduce hegemonic patriarchal patterns or promote alternative ways of being a man in contemporary society.	12
12	Riordan^(49)^	To present a method and framework for analyzing video-based pedagogy.	42
13	Layton^(50)^	To understand how students construct narratives about the presence of police in schools based on their experiences with School Resource Officers (SROs).	17
14	Johnston, et al.^(51)^	To develop a substantive grounded theory to explain how students experience teachers’ expectations of them.	59
15	Bonelli, et al.^(52)^	To understand families’ experiences in caring for children with myelomeningocele.	28
16	Riordan, et al.^(53)^	To clarify certain types of pedagogical problems encountered by teachers and students (presented as a Typology of Pedagogical Problems) and to understand and explain how participants sought to resolve these problems.	64
17	Vieira, et al.^(54)^	To understand the experience of maternal burden in caring for children with cancer from the mother’s perspective.	6
18	Forsberg^(55)^	To explore how children aged 11 to 15 talk about their social worlds and social incidents such as bullying.	36
19	Thornberg, et al.^(56)^	To examine in detail how a small sample of secondary school students explains why bullying occurs and to develop a grounded theory analysis based on students’ perspectives.	17
20	Vieira, et al.^(57)^	To understand families’ experiences of having a child using a cochlear implant (CI).	32
21	Faria, et al.^(58)^	To understand the meaning attributed by families to the family reintegration process and to construct a theoretical model representing this experience.	20
22	Forsberg^(59)^	To explore perspectives of 40 Swedish girls in 7th and 8th grade regarding bullying.	40
23	Salazar-Blanco, et al.^(60)^	To recognize the formative and transformative process of pediatric practice among professors in the Childhood Area of the Faculty of Medicine at the University of Antioquia.	19
24	Forsberg, et al.^(61)^	To explore how and in what ways children discuss bullying, in order to expand our knowledge of which processes are important to them.	48
25	Gomes, et al.^(62)^	To understand the meanings attributed by family caregivers in the hospital setting to their interactions with nursing staff.	15
26	Babler, et al.^(63)^	To gain a deeper understanding of adolescents’ experiences living with type 1 diabetes mellitus (T1DM) and to develop a theoretical paradigm.	11
27	Shannon, et al.^(64)^	To explore the perspectives of 21 preschool teachers in the United States regarding the professional development (PD) they received.	21
28	Leksell, et al.^(65)^	To increase knowledge about how children diagnosed with juvenile idiopathic arthritis (JIA) perceive their oral health and dental care.	15
29	Andrade, et al.^(66)^	To understand the meaning of caring for a child from the perspective of an underage adolescent mother, to uncover care-related demands, and to develop a theoretical model representing this experience.	9
30	Mieto, et al.^(67)^	To understand how mothers interpret the experience of accompanying their child in a Pediatric Hemodialysis Unit and to develop a theoretical model representing this experience.	11
31	Dupas, et al.^(68)^	To understand fathers’ experiences of having a child with cancer and to identify significant changes the illness brought to their perception of fatherhood.	5
32	Vieira, et al.^(69)^	To portray the family experience at the time of discovering a child’s hearing impairment.	32
33	Mendes, et al.^(70)^	To understand how pediatric nurses perceive the process of building partnerships with parents in the health care setting and to identify the actions they develop in practice.	12
34	Mendes, et al.^(71)^	To describe mothers’ experiences, the meaning and importance they attribute to participating in their child’s care, and to identify interventions in which they were involved.	18
35	Nunes, et al.^(72)^	To develop a theoretical model regarding the experience of families of preschool- and school-aged children with Down syndrome.	21
36	Nunes, et al.^(73)^	To understand what it is like for families to have a child with Down syndrome among their members.	21
37	Souza, et al.^(74)^	To understand the meaning of having sickle cell anemia for children aged 3 to 12 years.	8
38	Haraldsson, et al.^(75)^	To generate a theoretical model of the underlying social processes that trigger stress in adolescent girls’ everyday lives.	14
39	Damião, et al.^(76)^	To understand how adolescents with type 1 diabetes mellitus experience their illness and cope with it in daily life.	10
40	Vargens, et al.^(77)^	To analyze adolescent’s decision concerning pregnancy, based on the meaning they attribute to the phenomenon.	16
41	Soares, et al.^(78)^	To describe the phenomenon of motherhood among women attending a mental health outpatient clinic.	10
42	Vieira, et al.^(79)^	To understand the lived experience of children with Chronic Renal Insufficiency and to analyze the meaning they attribute to this experience.	8
43	Mendes, et al.^(80)^	To understand family dynamics in the experience of pediatric liver transplantation and to identify family demands and resources.	9
44	Sano, et al.^(81)^	To investigate mothers’ perceptions regarding the involvement of non-resident fathers in low-income families.	83
45	Pinto, et al.^(82)^	To understand how adolescents with type 1 diabetes mellitus experience their illness.	8
46	Rossato, et al.^(83)^	To understand the meaning of families’ experiences of children’s pain resulting from juvenile rheumatoid arthritis and to develop a theoretical model representing this experience.	27
47	Nunes, et al.^(84)^	To identify the meanings generated by chronic illness (diabetes) within family dynamics.	29
48	Moreira, et al.^(85)^	To understand children’s experiences of living with illness.	12
49	Pinto, et al.^(86)^	To understand the meaning of interactions experienced by families during a child’s hospitalization, to identify family demands, and to develop a theoretical model representing this experience.	7
50	Damião, et al.^(87)^	To understand adolescents’ experience of having a chronic illness such as type 1 diabetes mellitus.	7
51	Castro, et al.^(88)^	To understand the meaning of interactions experienced during the postoperative period of postectomy and to identify coping strategies used throughout the surgical experience.	12
52	Nunes, et al.^(89)^	To understand what it is like for a mother to have a child with diabetes and her relationship with the child, the illness, and the treatment.	6
53	Moreira, et al.^(90)^	To understand the meaning attributed by children aged 7 to 12 to health and illness.	27
54	Pauli, et al.^(91)^	To explore nurses’ beliefs regarding humanized care in the Pediatric Intensive Care Unit (PICU).	5
55	Zem-Mascarenhas, et al.^(92)^	To understand the experience of institutionalized children.	30
56	Bousso, et al.^(93)^	To understand family dynamics in the experience of Pediatric Intensive Care Unit (PICU) hospitalization and to develop a theoretical model representing the family’s experience.	24
57	Damião, et al.^(94)^	To understand how families experience periods of difficulty imposed by a child’s chronic illness.	8
58	Oliveira, et al.^(95)^	To understand the interactions experienced, to identify meanings attributed by accompanying family members during a child’s hospitalization, to identify the impacts of this experience on their lives, and to develop a theoretical model representing this experience.	18
59	Woodgate^(96)^	To obtain detailed descriptions of adolescents’ experiences of chronic illness.	23
Total			1166

In 24 (40.67%) studies, family members were the focus of scientific investigation, comprising a total of 416 participants, with mothers predominating in 22 (37.28%) publications. Only 12 (20.33%) studies investigated professionals, of which 7 (11.86%) focused predominantly on the Health field, 6 (10.16%) on Nursing professionals, and 5 (8.47%) on Education professionals.Adolescents participated in 29 (49.15%) studies. Of these, 24 (40.67%) specified the participants’ ages. Among the 27 (45.76%) articles that specified adolescents’ gender, 25 (42.37%) focused on girls. Children participated in only 15 (25.42%) studies, totaling 209 participants aged 3 to 9 years, with boys included in 15 (25.42%) and girls in 12 (20.33%) studies. 

Regarding the application of the concept of “Symbolic Interactionism”, Blumer was the most frequently cited theorist, mentioned in 25 (42.37%) articles, followed by Mead (22 articles). The most frequently cited work was “*Symbolic Interactionism: Perspective and Method*”, referenced in 25 (42.37%) articles. The six core concepts of SI were applied in the results, discussions, and final considerations of the studies, with social interaction appearing in 52 (88.13%) articles, society in 41 (69.49%), symbol in 39 (66.10%), self in 27 (45.76%), structure in 19 (32.20%), and mind in 7 (11.86%) texts.

Concerning the concept of “Grounded Theory”, 57 (96.61%) articles cited at least one scientific source as a reference. Strauss was the most frequently cited author, appearing in 40 (67.79%) articles, followed by Glaser in 27 (45.76%), Corbin in 21 (35.59%), and Charmaz in 12 (20.33%). Among the 53 (89.83%) studies that used books to guide methodological procedures, “*The Discovery of Grounded Theory: Strategies for Qualitative Research*” was the most influential work, cited in 37.28% of the articles. 

Regarding study design, 58 (98.30%) were qualitative studies, and only 1 (1.69%) adopted a mixed-methods approach. The predominant data collection instruments were semi-structured interview guides in 44 (74.57%) studies; open/in-depth interviews in 6 (10.16%); therapeutic play in 2 (3.37%); and video recordings in 2 (3.37%). In 39 (66.10%) articles, interviews were the sole data collection strategy. In the remaining studies, interviews were combined with two or more additional strategies/techniques: 5 (8.47%) used focus group interviews; 1 (1.69%) used the drawing-story technique; and 1 (1.69%) implemented an intervention. Additionally, 15 (25.42%) studies combined interviews with observation/participant observation techniques. 

Only 28 (47.45%) articles reported the data collection period, which ranged from two to 36 months; in 17 (28.81%) studies, data collection lasted 10 months or longer. Regarding the duration of interview recordings, only 34 (57.62%) articles reported this information. Specifically, 14 (23.72%) studies indicated a minimum duration of 10 minutes and a maximum of 100 minutes; some specified the exact total recording time, with a minimum of 388 minutes and a maximum of 990 minutes. 

Besides, only 27 (45.76%) studies reported the formation of Sampling Groups (SG). Among these, 18 (30.50%) included up to three SG; 7 (11.86%) included four to six sampling groups; and 2 (3.38%) included more than seven SG. Only 14 (23.72%) studies reported the justification for changes in SG, and just 3 (5.08%) specified the number of codes generated [12,001^48^, 21,436^43^ and 21,612^53^].

As analytical strategies, 23 (38.98%) studies mentioned the use of memos, and 25 (42.37%) presented diagrams. Only 7 (11.86%) articles used software for data analysis, and 3 (5.08%) used NVivo. Similarly, theory validation was conducted in 14 (23.72%) studies, including 5 (8.47%) theoretical model presentations; 2 (3.38%) interviews; 2 (3.38%) discussions; 2 (3.38%) guides/protocols; and 1 (1.69%) focus group.

Of the 27 (45.76%) studies conducted in health services, hospital care and specialized care were data collection settings in 16 (27.11%) and 11 (18.64%) studies, respectively. Among the 29 (48.27%) studies conducted in other contexts, 16 (27.11%) were carried out in schools; 2 (3.38%) in social programs; 1 (1.69%) in a support group; 1 (1.69%) in a religious institution; and 1 (1.69%) in an association.

## Discussion

This study demonstrated that research articles using GT and SI primarily focus on adolescents and family members as study populations. The theoretical-methodological application of Blumer’s and Strauss’s proposals stands out as the main theoretical and methodological frameworks, respectively. There was a predominance of interviews as the sole data collection strategy, as well as limited use of analytical resources such as memos, diagrams, and software. Theory validation occurred in few studies. Research was conducted predominantly in Southeastern Brazil and, to a lesser extent, in hospital and specialized healthcare settings.

The selected studies addressed therapeutic interventions, health conditions, diseases, and disorders. This aligns with social interests, as child and adolescent health is a research field that underpins several national and international public policies. Moreover, the Sustainable Development Goals (SDGs) intersect with these policies by directing child and adolescent needs based on scientific evidence^19^ ^,^ ^29^ ^-^ ^30^ ^,^ ^97^ ^-^ ^99^.

This reality stems from the impacts that health conditions, diseases, and disorders have on children. Through interactions, these conditions affect caregivers individually; however, their incidence is also observed in the broader health, social, and economic context of a country, as well as within the international community^13^ ^,^ ^98^ ^-^ ^100^. 

Seeking solutions and ensuring the resolution of these problems represents a challenge for science, to which research holds a social commitment^101^. For this reason, “Child” was the most frequently used descriptor in the studies. Despite this, and somewhat paradoxically, adolescents were the most frequently interviewed social group in the selected sample.

Adolescence is considered a complex phase, as individuals aged between 10 and 18 years^29^, in addition to undergoing bodily transformations, begin to be inserted into social relationships in which behavioral responses involving responsibility and autonomy are required^19^ ^-^ ^20^ ^,^ ^23^, unlike those permitted to children^16^ ^,^ ^19^ ^-^ ^20^ ^,^ ^22^. Therefore, generating knowledge about problems affecting this social group contributes to health practices that address their specific demands.

The findings show that the family perspective was the most frequently investigated in the selected studies. In qualitative research, it is common to elucidate phenomena from the perspective of family members, especially when addressing health issues of socially vulnerable groups, including children, adolescents, older adults, and people with disabilities^10^ ^,^ ^102^ ^-^ ^104^. 

This may be related to the dependency status of these groups and the close coexistence between individuals and their families, which increases the frequency of interactions and favors the maintenance of relationships^2^. Thus, it can be inferred that this social group is particularly capable of elucidating phenomena related to the health of these individuals.

Blumer was the most frequently cited theorist in the selected studies. This may be attributed to his importance within this theoretical approach, considering his role in compiling the assumptions, concepts, and ideas proposed by his predecessor, Mead^1^ ^-^ ^2^ ^,^ ^7^. Notably, Blumer was responsible for organizing and systematizing SI, as described in “*Symbolic Interactionism: perspective and method*”^2^ ^,^ ^7^, the most cited work among the selected studies.

It is necessary to resort to the application of a theoretical framework, since this constitutes “the core of the researcher’s thinking”^105^. Due to this centrality, it is observed that the authors of the selected studies sought to maintain theoretical reasoning based on the seminal work of SI, whose content was extracted directly from its precursor, Mead^1^ ^-^ ^2^. Such practice is recommended^7^ ^,^ ^105^ ^-^ ^107^, as it enables interpretations grounded in the ideas of the original author. 

The application of a theoretical framework compatible with the research object is a recognized practice in the construction of scientific knowledge^105^. In the selected studies, in addition to SI contributing to researchers’ perspectives regarding the investigated objects, its philosophical origin intersects with that of GT^3^ ^,^ ^7^. Possibly, this alignment favors the weaving of interactionist works within GT studies^105^ ^,^ ^107^ ^-^ ^108^. 

Strauss’s methodological proposals guided the majority of the selected studies. Perhaps Strauss was the scholar who most refined the methodological resources of the theory, as observed in his partnerships with Kate Charmaz and Juliet Corbin, with whom he developed the analytical tool known as the “paradigmatic model”^3^ ^,^ ^7^. 

This mechanism, within the coding stage, allows the organization of data and was refined to comprise three elements: condition, action-interaction, and consequence, replacing what was previously known as the 5Cs: context, causal conditions, intervening conditions, strategies, and consequences^3^ ^,^ ^109^. According to scholars, the adoption of three components allows flexibility for researchers^7^ ^,^ ^109^ ^-^ ^110^, which possibly facilitates the adoption of this strand^109^. 

Currently, at least four strands of GT are recognized^7^ ^,^ ^109^ ^-^ ^110^. The majority of the selected texts use more than one GT framework for methodological guidance, in such a way that authors discuss the need to standardize the application of the method based on a single framework^108^, as evidenced by 70% of the selected texts.

It should be noted that the methodological prescriptions presented in books require time for the development of scientific work. In order to ensure data depth and methodological rigor, the inductive-deductive corpus of GT includes the simultaneity of data collection and analysis, with commitment to strategies such as: verification of other perspectives regarding the information; the search for theoretical support in various sources; bibliographic consultations; and discussions of subthemes emerging from the data^3^ ^,^ ^7^. 

This multifactorial analysis enables the construction of new hypotheses and the formulation of questions that may be posed to other social groups experiencing the investigated phenomenon^3^ ^,^ ^4^ ^,^ ^110^. This dynamic of GT aims to fill thematic gaps, confirm or refute hypotheses, or complement information in light of its evident scarcity, in order to attribute breadth and variety to the data.

Such elements contribute to deepening the understanding of the phenomenon, guide the researcher’s reflections, and lead them to “move around” the phenomenon with distinct (or similar) observations, experiences, and perspectives that enrich the concepts constituting it^3^ ^,^ ^5^ ^,^ ^103^ ^,^ ^110^. 

To this end, it is necessary to ensure the existence of distinct characteristics in each sampling group - an essential element for selecting subsequent groups, since the distinctions of the subsequent group in relation to the previous one contribute to covering gaps identified in the data. This intensive process of data collection and analysis requires experience in qualitative studies and time from the scientific researcher^3^ ^-^ ^4^ ^,^ ^6^ ^,^ ^110^ ^-^ ^111^, which explains why some scholars took ten months or longer to complete this process.

The interview was the sole data collection strategy in most of the selected studies. However, in the most recent work on GT^3^, the combination of data collection strategies/techniques is recommended. This is justified because the use of multiple techniques is favorable to the development of this type of research, as some facilitate the researcher’s immersion in the field, the approach to sensitive topics, or engagement with research participants, such as the observation technique (participant or non-participant)^3^ ^,^ ^24^ ^,^ ^108^. In addition, the drawing-story technique has been successfully applied to vulnerable groups and sensitive topics^3^ ^,^ ^24^ ^,^ ^110^ ^,^ ^112^ ^-^ ^114^.

Thus, the use of multiple data collection methods must be compatible with the specific characteristics found in each research setting and each social group investigated^3^ ^,^ ^110^. It is also noted that observation contributes to gathering data inherent to the daily interactions of social actors and may reveal to the researcher realities that were not expressed during interviews^24^ ^,^ ^103^ ^,^ ^110^, but that become perceptible through the researcher’s coexistence with the group studied, especially when the researcher is external to the research setting. 

Therefore, incorporating different data collection techniques favors the development of substantive and formal theories. Thus, researchers who adopt the GT method, supported by the philosophical lens of SI, are encouraged to invest in a variety of data collection techniques^3^, an approach that enriches the researcher’s experience in the field. 

According to studies^107^ ^-^ ^108^ ^,^ ^111^, diagrams and memos are used in most of the theses and dissertations analyzed, as they support the reflexivity required in GT^3^ ^,^ ^5^ ^,^ ^24^ ^,^ ^106^ ^,^ ^115^ ^-^ ^116^. Paradoxically, this review highlights the need to expand the use of these analytical resources, as more than half of the texts did not report their use. Furthermore, there is a scarcity of methodological studies that elucidate the paths taken by authors^103^, or that seek scientific gaps through examining the theoretical-methodological application of qualitative studies. 

Indeed, the importance of describing methodological pathways in complex studies such as GT is widely recognized^24^ ^,^ ^103^ ^,^ ^108^. However, researchers often encounter publication rules that, in addition to challenging their ability to synthesize, increasingly limit the detailed reporting of methodological procedures in articles, given the requirement to qualitatively report the methodological conduct employed^117^, thus compelling authors to present the entire research process in a restricted manner.

This reality is identified in a study describing the process of theoretical sampling in the development of a Straussian GT, elucidating the explanations/information used in the primary study that guided the selection of sampling groups and, subsequently, culminated in theoretical sampling^103^. In GT, such choices must be explained/justified and be fundamentally related to the data^3^ ^,^ ^7^ ^,^ ^106^ ^,^ ^110^ ^-^ ^111^.

Few software programs are used in the analytical process, as only 7 (11.86%) texts reported using a computer program at this stage. A review of 239 studies highlights the predominance of software use in the analytical stage of qualitative research in theses and dissertations. Despite the difficulties reported regarding the use of this resource, researchers describe its potential benefits, such as optimizing the analysis time of large volumes of material and enabling the establishment of analytical patterns^118^, conditions advisable in GT studies.

NVivo was the most frequently used resource, although another study reports the existence of 11 different software programs available for qualitative research^118^. It is inferred that NVivo’s use stems from its longevity and popularity among researchers, as it is currently in its 15th version, suggesting broader diffusion among scholars. In summary, NVivo facilitates data preparation for analysis, visualization, and report generation^3^ ^,^ ^115^. 

This software is capable of importing various types of files and provides access to statistical data, textual data, and digital visual resources that may be used as data^3^ ^,^ ^115^. In other words, it enables the analysis of a variety of information, as required by some qualitative studies^115^ ^-^ ^116^, particularly GT^3^ ^,^ ^7^ ^,^ ^119^ ^-^ ^120^. Scholars agree that the use of software enhances research efficiency, as it assists in conducting the analysis^116^ ^,^ ^118^ ^,^ ^120^, in addition to contributing to methodological rigor by bringing collected data closer to the iterative returns to the field, a characteristic of GT^3^ ^,^ ^119^. 

During this period, the researcher remains immersed in the data, making it essential to condense a large volume of information^3^ ^,^ ^107^ ^,^ ^115^. The phases of coding, grouping, and the emergence of categories and subcategories make this analytical process intense^109^ ^,^ ^118^. The organization and storage of data are facilitated by the software^3^ ^,^ ^115^ ^-^ ^116^ ^,^ ^120^, which is beneficial and secure for the researcher, who must remain faithful to the raw data.

Studies warn of the scarcity of GT research in which theory validation is not presented in the methods section^107^, as also observed in the selected studies. Although not reported in all selected studies, validation of the emerging theory is essential for verifying its applicability, understanding, generalizability, and control, characteristics that allow breadth, depth, and variation of dimensions identified in the data and grounded in reality^3^ ^,^ ^4^ ^,^ ^110^. 

Furthermore, researchers acknowledge the need for theory validation, including in other social realities^3^ ^,^ ^7^ ^,^ ^110^. For this reason, in keeping with the method, some scholars validated the theory through discussions with methodological experts and specialists in the investigated topic^113^ ^,^ ^121^ ^-^ ^125^, while others incorporated an additional methodological approach for validation^119^ ^,^ ^126^ ^-^ ^127^ or confirmed the phenomenon through interviews with Swedish adolescents^128^. Thus, as suggested by the flexibility inherent in the method^3^ ^,^ ^110^, strategies for data collection, analysis, and validation can and should be adapted to the researcher’s possibilities and to the conditions encountered in the research field.

Finally, the most frequently investigated context was hospital health services, followed by specialized services. This may be because these are controlled environments in which researchers can easily observe groups of individuals who implicitly experience the same phenomenon. On the other hand, specialized care settings in emergency health situations constitute restricted-access structures that impose obstacles to data collection, especially through in-depth interviews, as experienced in qualitative studies conducted in hospitals in Northeastern Brazil^13^ ^,^ ^102^.

There is a clear absence of research conducted in countries in Africa, Central America, and Antarctica, while, within South America, Brazilian studies stood out. Consistent with another review^114^, GT research in the field of child and adolescent health has been concentrated in the Southeast and South regions, which host the largest number of universities and research centers in Brazil.

One limitation of this study is its inability to map research composed of children and adolescents as separate sampling groups, since these studies did not present results distinguished by age group, despite the specificity of each developmental stage. Nevertheless, this review resulted in a relevant screening that identified scientific gaps capable of motivating future research on latent themes. 

It also highlights the possibility of new qualitative reviews on topics of interest in the social and health fields. At the same time, it suggests the need for greater participation of children and professionals in future research to address the gaps identified here.

It is recommended that future studies address themes that fill the gaps related to the proposed theoretical-methodological application, in order to stimulate new and innovative scientific research grounded in SI under the methodological framework of GT. Finally, conducting similar reviews that include gray literature (especially theses and dissertations) is advisable for comparison with the results obtained.

## Conclusion

Adolescents and family members represent the majority of participants in the selected studies. Blumer and Strauss stand out as the main theoretical and methodological references, respectively, and most studies relied exclusively on interviews as the data collection strategy. Researchers in the selected studies made limited use of GT analytical resources, such as diagrams, memos, and software, as well as theory validation. Hospital and specialized services were the most frequently investigated contexts, with most studies conducted in Brazil. 

The scarcity of studies conducted with the simultaneous participation of children, adolescents, family members, and professionals constitutes a gap identified in this review. Furthermore, this study encourages the use of combined data collection strategies/techniques that enable researcher immersion in the field, foster closer engagement with the investigated group, and contribute to a broader representation of reality through data.

Attention is drawn to the need for greater use of analytical resources that contribute to methodological rigor, one of the gaps identified by this review. Additionally, studies conducted in states and countries with different sociocultural contexts are recommended in order to promote constant comparison. 

It is suggested that the scope of scientific investigations in the field of child and adolescent health be expanded using a similar theoretical-methodological framework, enabling interdisciplinary and/or interprofessional engagement with professionals, with a view to developing and deepening knowledge that contributes to the construction of substantive theories.

## Data Availability

The dataset of this article is available under the DOI 10.6084/m9.figshare.30675572.
