# Supplementary material for: Symbolic Interactionism and Grounded Theory in research on the health of children and adolescents: a scoping review
Source: Rev Lat Am Enfermagem. 2026 Oct 9;34:e4933. doi: 10.1590/1518-8345.7900.4933 (PMC13652535; doi:10.1590/1518-8345.7900.4933)
Supplement: Translation

# Translation: Interacionismo Simbólico e Teoria Fundamentada em pesquisas sobre a saúde de crianças e adolescentes: revisão de escopo^*^

**DOI:** 10.1590/1518-8345.7900.4934

**Authors:** Marimeire Morais da Conceição, Climene Laura de Camargo, Maria Ribeiro Lacerda, Maria Carolina Ortiz Whitaker, Amâncio António de Sousa Carvalho, Kamylla Santos da Cunha, Ariane Teixeira de Santana, Telmara Menezes Couto, Eliana Rosa da Fonseca, Cristiane Cardoso de Paula

**Affiliations:** 1 Universidade Federal da Bahia, Salvador, BA, Brasil.; 2 Secretaria de Saúde da Bahia, Escola de Saúde Pública da Bahia Professor Jorge Novis, Salvador, BA, Brasil.; 3 Bolsista da Coordenação de Aperfeiçoamento de Pessoal de Nível Superior (CAPES), Brasil.; 4 Bolsista do Conselho Nacional de Desenvolvimento Científico e Tecnológico (CNPq), Brasil.; 5 Universidade Federal do Paraná, Curitiba, PR, Brasil.; 6 Universidade de Trás-os-Montes e Alto-Douro, Escola Superior de Saúde, Quinta dos Prados, Vila Real, Portugal.; 7 Centro de Investigação em Estudos da Criança, Universidade do Minho, Braga, Portugal.; 8 Universidade Federal de Santa Catarina, Florianópolis, SC, Brasil.; 9 Governo Municipal, Secretaria Municipal de Saúde de Biguaçu, Biguaçu, SC, Brasil.; 10 Universidade Federal do Rio de Janeiro, Rio de Janeiro, RJ, Brasil.; 11 Universidade Federal da Santa Maria, Santa Maria, RS, Brasil.; 12 Instituto JBI, Centro Colaborador, São Paulo, SP, Brasil.

**Keywords:** Interacionismo Simbólico, Teoria Fundamentada, Saúde do Adolescente, Criança, Revisão, Saúde.

## Abstract

**Objetivo:**

mapear a aplicação teórico-metodológica do Interacionismo Simbólico e da Teoria Fundamentada em pesquisas sobre a saúde de crianças e adolescentes.

**Método:**

revisão de escopo conduzida conforme as recomendações do JBI. Consideraram-se: Participantes - crianças e/ou adolescentes, familiares e profissionais; Conceitos - Interacionismo Simbólico e Teoria Fundamentada nos Dados; Contexto - não delimitado. Foram consultadas nove fontes eletrônicas, incluindo artigos originais, sem restrição idiomática ou temporal. Para seleção e síntese dos resultados, utilizaram-se os *softwares* Rayyan e NVivo.

**Resultados:**

foram selecionados 59 estudos. A maioria dos participantes eram adolescentes (49%) e familiares (40%). Quanto à perspectiva teórica, 42% utilizaram o referencial de Blumer e 67% a metodologia de Strauss. A combinação de estratégias para coleta de dados ocorreu em 34% dos estudos; recursos analíticos, como diagramas e memorandos, foram citados em 42% e 39% dos estudos, respectivamente. Apenas 24% das pesquisas validaram a teoria. O contexto hospitalar foi o mais investigado (27%).

**Conclusão:**

a combinação de estratégias para coleta de dados possibilita maior variedade de análises, assim como investigações em diferentes contextos socioculturais e no campo intersetorial, com incremento de informações na construção de teorias substantivas. Recomendam-se estudos futuros com participação simultânea de crianças, adolescentes, familiares e profissionais.

## Destaques

**(1)** Adolescentes e familiares são os grupos sociais mais investigados.

**(2)** As obras de Strauss são as mais citadas como referencial metodológico.

**(3)** A combinação de estratégias para coleta de dados é um recurso pouco utilizado.

**(4)** Recursos analíticos, como diagramas e memorandos, são pouco utilizados.

**(5)** Menos de um quarto das pesquisas menciona a validação da teoria substantiva.

## Introdução

O Interacionismo Simbólico (IS) é uma teoria da Sociologia que surgiu no final do século XVII e foi impulsionada na década de 1930. Nesse processo, os estudiosos visavam explicar a relação entre as pessoas e a sociedade por meio das interações, bem como as implicações dessas interações no psiquismo dos indivíduos^1^ ^-^ ^2^. O IS parte de três pressupostos: 1) os seres humanos agem em relação às coisas/situações com base nos significados que lhes atribuem; 2) o significado dessas coisas/situações origina-se a partir das interações em sociedade; e 3) tais significados são transformados por meio das interpretações da pessoa ao lidar com elas. Em meio a esses pressupostos, o IS baseia-se em seis conceitos: sociedade, símbolo, mente, *self,* estrutura e interação social^2^.

Assim, o IS introduz discussões sobre os conceitos do ser social, cujas bases teóricas estão ancoradas nas ideias de George Herbert Mead, seguido durante anos pelo também sociólogo Herbert Blumer, que reuniu, organizou, estruturou e registrou em escritos os pensamentos de Mead^1^ ^-^ ^2^. Esse conteúdo era apresentado em aulas, reuniões e palestras na Universidade de Chicago, onde se formou Anselm Strauss^2^ ^-^ ^3^. 

Strauss recebeu influências do IS e, juntamente com outro sociólogo, Barney Glaser, desenvolveu a Teoria Fundamentada nos Dados (TFD)^3^. A TFD é uma metodologia de pesquisa que segue o pensamento positivista. Entretanto, Strauss acrescentou formas analíticas interpretativistas que dialogam com o IS^2^ ^-^ ^3^, dando origem às raízes teóricas da TFD na pesquisa qualitativa^3^.

No emprego da TFD, o pesquisador busca evidenciar estratégias desenvolvidas pelos indivíduos ao lidar com diversos objetos sociais, especialmente situações vivenciadas em seu contexto de vida, juntamente com a sociedade da qual fazem parte^3^ ^-^ ^5^. Por esse motivo, a aplicação da TFD é reconhecida atualmente pelo êxito em pesquisas que objetivam gerar teorias formais e substantivas^3^ ^-^ ^6^. Dando continuidade ao propósito inicial de Glaser e Strauss, o método mostra-se adequado para abordar temáticas e processos sociais considerados difíceis e multifacetados^3^ ^,^ ^7^, assim como o fazem as pesquisas da área da saúde. 

Salienta-se que, no presente estudo, o termo saúde condiz com o conceito cunhado pela Organização Mundial da Saúde (OMS), ampliado multidimensionalmente. Portanto, abarca não somente o campo biológico, comportamental e socioeconômico, mas também acrescenta ao escopo condições ambientais e relações políticas^8^ ^-^ ^9^. Estudiosos consideram as interações essenciais na realização de ações em prol da saúde de crianças e adolescentes^10^ ^-^ ^13^. Logo, torna-se relevante centrar a aplicação teórico-metodológica da TFD associada ao IS em investigações científicas sobre a saúde dessa população, uma vez que esses dois referenciais favorecem a apreensão das relações estabelecidas entre os indivíduos na sociedade.

Essa experiência social inicia-se no intraútero^14^ ^-^ ^15^, é conduzida na infância e adolescência pela família (socialização primária) e perdura ao longo da vida com a participação de profissionais da saúde e da educação (socialização secundária), como mostram estudos realizados no Brasil, na China, na Holanda e nos Estados Unidos da América (EUA)^11^ ^-^ ^12^ ^,^ ^16^ ^-^ ^17^. No contexto atual de reconhecimento de direitos, torna-se cada vez mais comum que crianças e adolescentes tenham informações sobre si e sobre sua condição de saúde^18^ ^-^ ^21^, cenário que permite a participação na tomada de decisão e no autocuidado, em observância à autonomia e ao protagonismo social infantojuvenil^20^ ^,^ ^22^ ^-^ ^24^, elementos que se contrapõem ao cuidado adultocentrado^19^ ^,^ ^21^ ^,^ ^25^. 

Este estudo aponta vazios científicos que poderão ser usados para justificar a realização de novos estudos com temáticas latentes, a partir da utilização de estratégias de coleta de dados e da aplicação de recursos analíticos voltados ao desenvolvimento de teorias. Constitui, também, um compilado da aplicação teórico-metodológica do IS e da TFD nas pesquisas, além de incentivar o incremento de estudos em contextos socioculturais inovadores e inéditos. Por fim, o objetivo desta revisão foi mapear a aplicação teórico-metodológica do Interacionismo Simbólico e da Teoria Fundamentada em pesquisas sobre a saúde de crianças e adolescentes.

## Método

### Tipo de estudo 

Revisão de Escopo (RE) baseada nas recomendações propostas pelo JBI^26^ ^-^ ^27^ ^)^ e apresentada conforme o *checklist* PRISMA *Extension for Scoping Reviews* (PRISMA-ScR)^28^. O protocolo foi registrado no *Open Science Framework* (OSF): https://doi.org/10.17605/OSF.IO/BKTMJ. 

A equipe de revisão foi composta por três profissionais com experiência teórico-metodológica em IS e TFD, sendo uma revisora com conhecimento na área de saúde da criança e do adolescente, uma consultora metodológica com expertise em revisão de escopo pelo método JBI e uma profissional bibliotecária com expertise em estudos de revisão na área da saúde.

### Questão de revisão 

Foi utilizado o mnemônico PCC^27^, que representa: (P) População - crianças, adolescentes, familiares e/ou profissionais; (C) Conceito - IS e TFD; e (C) Contexto - qualquer ambiente. A partir disso, a pergunta norteadora foi formulada da seguinte forma: como são aplicados, teórico-metodologicamente, o IS e a TFD nas pesquisas sobre saúde de crianças e adolescentes? 

Foram elencadas questões secundárias para condução do mapeamento: Quais grupos sociais são abordados em estudos sobre saúde da criança e do adolescente? Quais ferramentas analíticas e metodológicas são utilizadas na produção do conhecimento? 

### Acrônimo PCC

Foram eleitos como *participantes*: 1) crianças e adolescentes: idade de dois a 18 anos incompletos, seguindo o referencial da Organização Mundial da Saúde (OMS)^29^; 2) cuidadores familiares: pessoas que detêm o poder legítimo sobre a criança e/ou adolescente, tenham relação familiar e/ou mantenham (ou não) parentesco com a criança ou adolescente, como pai/mãe, padrasto/madrasta, avô(ó), tio(a), irmão(ã), primo(a)^29^ ^-^ ^31^; 3) cuidadores não familiares: pessoas que cuidam de crianças e/ou adolescentes, embora não sejam membros da família nem profissionais da saúde, como babás, professores(as), supervisores(as), treinadores(as)^29^ ^,^ ^32^; 4) profissionais da saúde: indivíduos com formação na área da saúde, como equipe de enfermagem, médico(a), nutricionista, fisioterapeuta, fonoaudiólogo(a), assistente social, psicólogo(a), terapeuta ocupacional, Agentes Comunitários de Saúde (ACS)^29^ ^-^ ^30^ ^,^ ^33^; e 5) profissionais sem formação em saúde, como educadores e agentes de proteção e segurança infantojuvenis^30^. 

No *conceito*, foram incluídos exclusivamente os estudos que investigam as formas de aplicação teórico-metodológica do IS nas pesquisas conduzidas pelo método TFD. O IS afirma que os indivíduos reagem aos objetos sociais (objetos, falas, ações, pessoas), recorrendo a símbolos e atribuindo-lhes significados^1^ ^-^ ^2^.

A TFD é uma metodologia da pesquisa científica que orienta a construção de teorias por meio do agrupamento de conceitos. Para isso, recorre à coleta e à análise simultâneas, rigorosamente sistematizadas para evidenciar conceitos e demonstrar como eles se relacionam entre si e em torno de um fenômeno central. A teoria emergente a partir dos dados explica as experiências dos indivíduos e justificam suas reações diante dos acontecimentos com os quais se deparam^3^ ^,^ ^7^. 

Admitiu-se qualquer contexto nacional ou internacional, considerando qualquer cenário de atenção à saúde da criança e do adolescente. 

Utilizaram-se como critérios de inclusão: artigos publicados em revistas ou periódicos revisados por pares, às cegas, provenientes de pesquisa de campo, estudos originais e empíricos, de qualquer abordagem, embasados no IS, cuja condução metodológica se deu a partir da TFD. Devido à possibilidade de duplicação de dados extraídos dos métodos dos estudos selecionados, não foram admitidas literaturas cinzentas. Foram excluídos os estudos com mais de um referencial teórico-metodológico. 

### Coleta dos dados

A estratégia de busca foi elaborada pela bibliotecária que integrou a equipe desta revisão. A partir do acrônimo, identificaram-se os termos de busca nos vocabulários controlados - Descritores em Ciências da Saúde (DeCS), *Medical Subject Heading* (MeSH) e *Embase Subject Headings* (Emtree) - e realizaram-se duas buscas preliminares. Após a definição da estratégia de busca (Figura 1), esta foi adaptada a cada base de dados.

**Figura 1:** Estratégia de busca aplicada à MEDLINE/PubMed em 08 de fevereiro de 2023, atualizada em 10 de julho de 2024

Busca	Estratégia de Busca	Resultados
#1	Search: “Grounded Theory”[mh] OR Grounded Theory[tiab] Sort by: Most Recent	14,791
#2	Search: “Symbolic Interactionism”[mh] OR Symbolic Interactionism*[tiab] OR symbolic interaction[tiab] OR symbolic interaction theory[tiab] OR “Social interaction”[mh] OR Social Functioning*[tiab] OR Social Interaction*[tiab] Sort by: Most Recent	40,810
#3	Search: “Infant”[mh] OR Infant*[tiab] OR “Child, Preschool”[mh] OR Preschool*[tiab] OR “Child”[mh] OR Child*[tiab] OR Children[tiab] OR Pediatric*[tiab] OR “Adolescent[mh]” OR Adolescence[tiab] OR Adolescent*[tiab] OR Teen*[tiab] OR Teenager*[tiab] OR Youth*[tiab] OR “Child Health”[mh] Sort by: Most Recent	3,519,518
#4	Search: (#1 AND #2) AND #3 Sort by: Most Recent	84

As buscas foram realizadas nas seguintes fontes: MEDLINE (PubMed), *Scientific Electronic Library Online* (SciELO), Banco de Dados de Enfermagem (via Portal da Biblioteca Virtual em Saúde), *Cumulative Index to Nursing and Allied Health Literature* (CINAHL), *Education Resources Information Center (ERIC),* *Fonte Acadêmica da Scopus (EBSCO), Embase (Elsevier), Web of Science* (*Clarivate Analytics*) e *PsycINFO*.

Inicialmente, as 1.357 referências recuperadas foram importadas no gerenciador EndNote para identificação e exclusão de duplicatas e, em seguida, exportadas para o Rayyan, programa *web* gratuito financiado pela Qatar Foundation^34^. A seleção dos textos foi conduzida de forma duplamente independente, a partir dos critérios de inclusão. A primeira triagem ocorreu por meio da análise de título e resumo; a segunda, pela leitura do texto na íntegra. Quando houve dissenso entre as revisoras, uma terceira pessoa foi consultada para a tomada de decisão^27^. 

As 1.947 referências foram analisadas após a seleção de 51 estudos, e oito textos foram adicionados. Nove textos não encontrados na íntegra foram solicitados aos autores, via correio eletrônico; porém, somente um autor disponibilizou o texto completo, que foi incorporado aos resultados.

A revisora 1 confeccionou uma planilha no Excel e uma legenda contendo todas as variáveis e suas respectivas descrições, a fim de uniformizar a extração dos dados^35^. Um teste-piloto^27^ possibilitou os ajustes na planilha. Na etapa 1, extraíram-se dados como: autores, objetivo, tema, descritores, ano de publicação e país onde a pesquisa foi realizada.

Na etapa 2, extraíram-se os seguintes elementos: 1) participantes: amostra; participação de crianças e/ou adolescentes (número, idade, sexo, raça/cor); participação de familiares (número, idade, sexo, raça/cor, vínculo com a criança/adolescente); profissionais (número, idade, sexo, raça/cor, área de atuação); 2) conceitos: no IS, autores citados, obras citadas e aplicação dos seis conceitos; na TFD, autores, obra citada, abordagem metodológica, técnica/instrumento de coleta de dados, duração das entrevistas, período de coleta de dados, memorandos, diagramas, número de códigos extraídos, quantidade de grupos amostrais, *software* utilizado, validação e tipo de validação; e 3) contexto: local da investigação (serviços de saúde, educação, comunidade, associações), sendo os serviços de saúde foram classificados como hospitalar, especializado ou básico.

Para a extração de dados, procedeu-se à leitura atenta do material selecionado por duas revisoras independentes^27^ ^,^ ^35^ ^-^ ^36^, os quais foram agrupados, sintetizados no Microsoft Excel e, posteriormente, analisados no *software* NVivo versão 14. No Excel, os artigos receberam um identificador numérico de 01 a 59, de acordo com o ano e a ordem de publicação.

A presença das variáveis que compunham o PCC foi sinalizada na planilha de forma dicotomizada, com respostas de uma única palavra (como “sim” ou “não”) que foram submetidas à análise quantitativa básica^35^, gerando o percentual relativo ao total de artigos. A distribuição geoespacial dos textos publicados foi realizada com base em fórmula automática do Excel; considerando a frequência de realização dos estudos em diferentes países. A nuvem de palavras foi gerada a partir do agrupamento automático dos descritores/palavras-chave no NVivo. 

Recorreu-se à análise qualitativa descritiva básica de conteúdo^35^, na qual trechos dos textos foram selecionados, receberam o identificador numérico atribuído ao artigo de origem, foram transformados em códigos e agrupados por temas provenientes dos seis conceitos basais do IS^1^ ^-^ ^2^. Consecutivamente, procedeu-se à contagem da frequência desses elementos.

## Resultados

Foram selecionados 59 artigos, publicados entre 1998 e 2024, sendo 33 (55,93%) publicados em periódicos nacionais e 17 (28,81%), nos últimos cinco anos. Os resultados estão representados na Figura 2 pelo fluxograma *Preferred Reporting Items for Systematic reviews and Meta-Analyses* (PRISMA)^37^:

**Figura 2:** Fluxograma PRISMA^37^ de seleção dos estudos. Salvador, BA, Brasil, 2024

As pesquisas ocorreram em cinco continentes: 35 (59,32%) na América do Sul; 10 (16,94%) na Europa; 7 (11,86%) na América do Norte; 2 (3,38%) na Ásia; e 1 (1,69%) na Oceania. Na América do Sul, destacam-se 31 (52,54%) produções brasileiras, das quais 29 (49,15%) foram realizadas em estados do Sudeste, 1 (1,69%) na região Sul, e 2 (3,38%) não informam a região, estado ou município onde a pesquisa foi realizada. A distribuição geográfica das produções selecionadas está demonstrada na Figura 3:

**Figura 3:** Distribuição geoespacial dos estudos que apresentaram o local de pesquisa (n = 55). Salvador, BA, Brasil, 2024

Ao todo, 58 (98,30%) artigos apresentaram descritores extraídos do DeCS ou do MeSH. Observa-se, nos descritores usados, que o termo “Enfermagem” (e correlatos) foi utilizado em 30 (50,84%) artigos; o termo “Criança”, em 25 (42,37%) artigos; e a expressão “Teoria Fundamentada” em 11 (18,64%) artigos, dados congruentes com a Figura 4:

**Figura 4:** Nuvem de palavras gerada a partir dos descritores/palavras-chave dos artigos (n = 58). Salvador, BA, Brasil, 2024

Os autores investigaram temáticas que envolvem: doenças, 17 (28,81%); relações sociais, 13 (22,03%) - relacionadas às interações estabelecidas entre os indivíduos^2^; e processos sociais, 9 (15,25%) - respostas individuais ou coletivas frente aos objetos, situações e contextos sociais^2^. Além disso, 7 (11,86%) estudos relacionam-se a intervenções terapêuticas; 5 (8,47%) abordam agravos; 3 (5,08%) abordam síndromes; 3 (5,08%) tratam de transtornos; e 2 (3,38%) referem-se a deficiências.

Na Figura 5, os textos são apresentados da publicação mais recente para a mais antiga.

**Figura 5:** Síntese da caracterização dos artigos selecionados: autor, país, objetivo e amostra. Salvador, BA, Brasil, 2024

Nº	Autor	Objetivo	Amostra
1	Juan-Martínez, et al.^38^	Compreender as reflexões sobre a construção de identidades de gênero e as implicações da violência em adolescentes indígenas no nordeste de Oaxaca.	45
2	Queiroz, et al.^39^	Analisar os fatores intervenientes na gerência do cuidado de enfermagem à criança hospitalizada com cardiopatia reumática.	19
3	Mazaherizadeh, et al.^40^	Explorar os fatores subjacentes, os processos e as consequências das desordens por jogos entre os adolescentes iranianos do sexo masculino e os fatores contextuais relacionados com as desordens por jogos no âmbito da tapeçaria sociocultural e psicológica única do Irã.	13
4	Picolo Gimenes, et al.^41^	Compreender como os enfermeiros se representam ao vivenciar o uso do Jogo Terapêutico no cuidado de crianças e adolescentes com o apoio do Desenho-História.	11
5	Rafii, et al.^42^	Explorar o processo de vida das mães de crianças com autismo.	14
6	Novoa-Villegas, et al.^43^	Analisar os significados que meninos e meninas têm sobre a configuração da(s) masculinidade(s) expressa pela literatura infantil feminista.	10
7	Giraldo-Giraldo, et al.^44^	Indagar sobre a relação entre a apropriação e uso das redes sociais virtuais e a construção do si mesmo em adolescentes escolares.	19
8	Moreira-Dias, et al.^45^	Apresentar um modelo teórico sobre o contexto interacional entre profissionais de saúde e famílias de crianças e adolescentes em cuidados paliativos.	10
9	Gimenes, et al.^46^	Compreender como o enfermeiro significa seu papel na interação com a criança durante a utilização do Brinquedo Terapêutico e construir um modelo teórico representativo dessa vivência.	11
10	Sethi^47^	Aprofundar o conhecimento existente sobre as influências na paternidade por meio de uma lente ocupacional.	12
11	Pérez-Rivera, et al.^48^	Apresentar e discutir significados e práticas sobre a paternidade e a masculinidade que se dão na criação dos filhos e que levam à reprodução social dos padrões hegemônicos patriarcais ou à emergência de formas alternativas de ser homem na sociedade atual.	12
12	Riordan^49^	Apresentar um método e uma estrutura para análise de pedagogia baseada em vídeo.	42
13	Layton^50^	Entender como os alunos constroem narrativas sobre a presença da polícia em suas escolas com base em suas experiências com os agentes de recursos escolares (SROs).	17
14	Johnston, et al.^51^	Desenvolver uma teoria fundamentada substantiva para explicar como os alunos vivenciam as expectativas dos professores em relação a eles.	59
15	Bonelli, et al.^52^	Compreender a experiência de famílias no cuidado de crianças com mielomeningocele.	28
16	Riordan, et al.^53^	Esclarecer alguns dos tipos de problemas pedagógicos que os professores e alunos encontram (apresentados como uma Tipologia de Problemas Pedagógicos) e entender e explicar como os participantes procuraram resolver esses problemas.	64
17	Vieira, et al.^54^	Compreender a experiência da sobrecarga materna no cuidado dos filhos com câncer do ponto de vista da mãe.	6
18	Forsberg^55^	Explorar como as crianças de 11 a 15 anos de idade falam sobre seus mundos sociais e incidentes sociais, como *bullying*.	36
19	Thornberg, et al.^56^	Examinar, em detalhes, como uma pequena amostra de alunos do ensino médio explica por que o *bullying* acontece e desenvolver uma análise de teoria fundamentada com base nas perspectivas dos alunos.	17
20	Vieira, et al.^57^	Compreender a experiência da família de uma criança que usa um implante coclear (IC).	32
21	Faria, et al.^58^	Compreender o significado do processo da reintegração familiar atribuído pela família que o vivencia e construir um modelo teórico representativo dessa vivência.	20
22	Forsberg^59^	Explorar as perspectivas de 40 meninas suecas de 7ª e 8ª séries sobre o *bullying*.	40
23	Salazar-Blanco, et al.^60^	Reconhecer o processo formativo e transformador da prática pediátrica dos professores da Área da Infância da Faculdade de Medicina da Universidade de Antioquia.	19
24	Forsberg, et al.^61^	Explorar como e de que forma as crianças discutem o *bullying*, para ampliar nosso conhecimento sobre quais processos são importantes para as crianças.	48
25	Gomes, et al.^62^	Compreender os significados atribuídos por familiares cuidadores da criança no ambiente hospitalar acerca de suas interações com os profissionais da equipe de enfermagem.	15
26	Babler, et al.^63^	Obter uma maior compreensão das experiências dos adolescentes que vivem com diabetes mellitus tipo 1 (DM1) e criar um paradigma teórico.	11
27	Shannon, et al.^64^	Explorar as perspectivas de 21 professores de pré-escola, localizados nos Estados Unidos, sobre o desenvolvimento profissional (DP) que receberam.	21
28	Leksell, et al.^65^	Aumentar o conhecimento sobre como as crianças diagnosticadas com artrite idiopática juvenil (AIJ) percebem sua saúde bucal e atendimento odontológico.	15
29	Andrade, et al.^66^	Compreender o significado de cuidar de um filho para a mãe adolescente menor, desvelar as demandas para o cuidado e construir um modelo teórico sobre essa vivência.	9
30	Mieto, et al.^67^	Compreender como as mães significam a experiência de acompanhar o filho em uma Unidade de Hemodiálise Infantil e construir um modelo teórico representativo dessa experiência.	11
31	Dupas, et al.^68^	Compreender a experiência do pai de uma criança com câncer, identificando quais mudanças significativas a doença provocou na sua forma de encarar a paternidade.	5
32	Vieira, et al.^69^	Retratar a experiência familiar quando da descoberta da deficiência auditiva de um filho.	32
33	Mendes, et al.^70^	Compreender como os enfermeiros de pediatria veem o processo de formação de parceria com os pais na área da saúde e identificar as ações que desenvolvem no ambiente.	12
34	Mendes, et al.^71^	Descrever a experiência das mães, o significado e a importância que atribuem à sua participação no processo de cuidado de seus filhos, bem como identificar intervenções nas quais estavam envolvidas.	18
35	Nunes, et al.^72^	Desenvolver um modelo teórico sobre a experiência de famílias de crianças com síndrome de Down em idade pré-escolar e escolar.	21
36	Nunes, et al.^73^	Compreender como é para a família ter uma criança com Síndrome de Down entre seus membros.	21
37	Souza, et al.^74^	Compreender o significado de ter anemia falciforme para crianças de 3 a 12 anos de idade.	8
38	Haraldsson, et al.^75^	Gerar um modelo teórico dos processos sociais subjacentes que desencadeiam o estresse na vida cotidiana das adolescentes.	14
39	Damião, et al.^76^	Compreender como o adolescente com diabetes mellitus tipo 1 vivencia sua experiência de doença e como lida com essa situação no cotidiano.	10
40	Vargens, et al.^77^	Analisar a decisão da adolescente pela gravidez, com base no significado que ela atribuiu ao fenômeno.	16
41	Soares, et al.^78^	Descrever o fenômeno da maternidade para usuárias de um ambulatório de saúde mental.	10
42	Vieira, et al.^79^	Compreender a vivência da criança com insuficiência renal crônica e analisar o significado que ela atribui a essa vivência.	8
43	Mendes, et al.^80^	Compreender a dinâmica familiar na experiência do transplante hepático pediátrico e identificar as demandas e recursos da família.	9
44	Sano, et al.^81^	Investigar as percepções das mães sobre o envolvimento de pais não residentes em famílias de baixa renda.	83
45	Pinto, et al.^82^	Compreender como o adolescente com diabetes mellitus tipo 1 vivencia sua experiência de doença.	8
46	Rossato, et al.^83^	Compreender o significado da experiência da família da criança com dor decorrente de artrite reumatoide juvenil e construir um modelo teórico representativo dessa experiência.	27
47	Nunes, et al.^84^	Identificar os significados que a situação de doença crônica (diabetes) gera na dinâmica familiar.	29
48	Moreira, et al.^85^	Compreender a experiência da criança na vivência com a doença.	12
49	Pinto, et al.^86^	Compreender o significado das interações vivenciadas pela família durante a hospitalização de uma criança de seu núcleo, identificar as demandas da família e construir um modelo teórico representativo dessa experiência.	7
50	Damião, et al.^87^	Compreender a experiência do adolescente em ter uma doença crônica como o diabetes mellitus tipo 1.	7
51	Castro, et al.^88^	Compreender o significado das interações vivenciadas ante o pós-operatório de postectomia e identificar estratégias durante a experiência cirúrgica.	12
52	Nunes, et al.^89^	Compreender como é para a mãe ter um filho diabético; o relacionamento dela com criança, doença e tratamento.	6
53	Moreira, et al.^90^	Compreender o significado que a criança, na faixa etária de 7 a 12 anos, atribui à saúde e à doença.	27
54	Pauli, et al.^91^	Conhecer as crenças das enfermeiras em relação à assistência humanizada na Unidade de Terapia Intensiva pediátrica (UTIp).	5
55	Zem-Mascarenhas, et al.^92^	Conhecer a experiência da criança institucionalizada.	30
56	Bousso, et al.^93^	Compreender o funcionamento da dinâmica familiar da criança internada em UTI pediátrica e construir um modelo teórico sobre a experiência da família que vivencia a internação da criança na UTI pediátrica.	24
57	Damião, et al.^94^	Compreender como a família vivencia os períodos de dificuldades impostos pela doença crônica da criança.	8
58	Oliveira, et al.^95^	Compreender as interações vivenciadas, identificar os significados que o familiar acompanhante atribui à experiência de vivenciar a hospitalização da criança, identificar o que essa vivência provoca na vida do familiar e construir um modelo teórico representativo da experiência.	18
59	Woodgate^96^	Obter descrições detalhadas das experiências de doenças crônicas dos adolescentes.	23
Total			1166

Em 24 (40,67%) estudos, os familiares foram alvo de investigação científica, compondo o grupo de 416 participantes, com predominância de mães em 22 (37,28%) produções. Apenas 12 (20,33%) investigaram profissionais, sendo 7 (11,86%) com predominância da área da Saúde, 6 (10,16%) sobre profissionais da Enfermagem, e 5 (8,47%) da Educação.A participação de adolescentes ocorreu em 29 (49,15%) estudos. Destes, 24 (40,67%) especificam a idade dos adolescentes participantes. Dos 27 (45,76%) artigos que especificaram o gênero dos adolescentes participantes, 25 (42,37%) pesquisaram meninas. Apenas em 15 (25,42%) dos textos crianças participaram da pesquisa, totalizando 209 participantes com idade entre 3 e 9 anos, com participação de meninos em 15 (25,42%) e de meninas 12 (20,33%) estudos. 

Na aplicação do conceito “Interacionismo Simbólico”, o teórico mais estudado foi Blumer, mencionado em 25 (42,37%) artigos, seguido de Mead (22 artigos). A obra mais frequentemente citada foi “*Symbolic Interactionism: perspective and method*”, citada em 25 (42,37%) artigos. Os seis conceitos do IS foram aplicados nos resultados, discussões e considerações finais dos estudos, prevalecendo as interações sociais em 52 (88,13%), sociedade em 41 (69,49%), símbolo em 39 (66,10%), *self* em 27 (45,76%), estrutura em 19 (32,20%) e mente em 7 (11,86%) textos.

Quanto ao conceito “Teoria Fundamentada nos Dados”, 57 (96,61%) textos citaram ao menos um material científico como referência. O autor mais citado foi Strauss em 40 (67,79%) textos, seguido de Glaser em 27 (45,76%), Corbin em 21 (35,59%) e Charmaz em 12 (20,33%). Nos 53 (89,83%) artigos que utilizaram livros na condução metodológica, evidenciou-se que “*The Discovery of grounded theory: Strategies for qualitative research*” foi a obra que mais orientou os autores, presente em 37,28% dos textos. 

Quanto à abordagem, 58 (98,30%) foram estudos qualitativos e apenas 1 (1,69%) foi quanti-qualitativo. Predominaram como instrumentos de coleta de dados: 44 (74,57%) roteiros de entrevistas semiestruturadas; 6 (10,16%) entrevistas abertas/em profundidade; 2 (3,37%) brinquedos terapêuticos; e 2 (3,37%) filmagens. Em 39 (66,10%) artigos, a entrevista foi a única estratégia de coleta de dados. Nos demais textos, além da entrevista, foram usadas duas ou mais estratégias/técnicas: 5 (8,47%) entrevistas em grupo/grupo focal; 1 (1,69%) desenho-história; e 1 (1,69%) intervenção. Ainda, 15 (25,42%) estudos agregaram à entrevista a técnica de observação/observação-participante. 

Somente 28 (47,45%) textos informam o período de coleta de dados, que variou entre dois e 36 meses, sendo que, em 17 (28,81%) estudos, o tempo de coleta de dados foi igual ou superior a 10 meses. Quanto ao tempo de gravação das entrevistas, apenas em 34 (57,62%) textos registraram esse dado. Especificamente, 14 (23,72%) estudos mencionam duração mínima de 10 minutos e máxima de 100 minutos; alguns especificaram exatamente o tempo total de gravação, com mínimo de 388 minutos e máximo de 990 minutos. 

Ademais, apenas 27 (45,76%) estudos mencionaram a formação de Grupos Amostrais (GA). Dentre os artigos, 18 (30,50%) apresentaram até três GA; 7 (11,86%) de quatro a seis grupos amostrais; e 2 (3,38%) acima de sete GA. Apenas 14 (23,72%) estudos registram as justificativas para mudança de GA, e somente 3 (5,08%) mencionaram o número de códigos utilizados [12.001^48^, 21.436^43^ e 21.612^53^].

Como estratégia analítica, 23 (38,98%) estudos mencionaram o uso de memorandos/memos, e 25 (42,37%) apresentaram diagramas. Somente 7 (11,86%) artigos utilizaram *softwares* para análise, e 3 (5,08%) estudos usaram o NVivo. De forma semelhante, a validação da teoria foi realizada em 14 (23,72%) estudos, com 5 (8,47%) apresentações do modelo teórico; 2 (3,38%) entrevistas; 2 (3,38%) conversas; 2 (3,38%) guias/protocolos e 1 (1,69%) grupo focal.

Do total de 27 (45,76%) pesquisas realizadas em serviços de saúde, a Atenção Hospitalar e a Atenção Especializada foram campos de coleta de dados em 16 (27,11%) e 11 (18,64%) estudos, respectivamente. Dos 29 (48,27%) estudos realizados em outros contextos, 16 (27,11%) ocorreram em escolas; 2 (3,38%) em programas sociais; 1 (1,69%) em um grupo de apoio; 1 (1,69%) em uma instituição religiosa; e 1 (1,69%) em uma associação.

## Discussão

Este estudo evidenciou que os artigos de pesquisas que utilizam a TFD e o IS têm como população adolescentes e familiares. Destaca-se a aplicação teórico-metodológica das propostas de Blumer e Strauss, respectivamente, como referencial teórico e metodológico. Observa-se predominância da utilização da entrevista como única estratégia de coleta de dados, bem como uso limitado de recursos analíticos, como memorandos, diagramas e *softwares*. A validação da teoria ocorreu em poucos estudos. As pesquisas ocorreram majoritariamente no Sudeste brasileiro e, em menor proporção, em contextos hospitalares e especializados da saúde.

Os estudos selecionados abordam intervenções terapêuticas, agravos, doenças e transtornos. Isso coaduna com os interesses sociais, pois a saúde da criança e do adolescente é um campo de pesquisa que embasa diversas políticas públicas nacionais e internacionais. Além disso, os Objetivos do Desenvolvimento Sustentável (ODS) tangenciam essas políticas, direcionando necessidades infantojuvenis com base em evidências científicas^19^ ^,^ ^29^ ^-^ ^30^ ^,^ ^97^ ^-^ ^99^.

Tal realidade decorre dos impactos que afetam crianças em decorrência de agravos, doenças e transtornos. Essas condições, por meio das interações, repercutem individualmente em seus cuidadores; porém, também se observam incidências no cenário geral de saúde, social e financeiro de um país, bem como perante a comunidade internacional^13^ ^,^ ^98^ ^-^ ^100^. 

Buscar soluções e garantir a resolução desses problemas consiste em um desafio para a ciência, com a qual as pesquisas assumem compromisso social^101^, motivo pelo qual “Criança” foi o descritor mais utilizado nos estudos. Apesar disso, e de forma contraditória, os adolescentes foram o grupo social mais entrevistado na amostra selecionada.

A adolescência é considerada uma fase complexa, pois o indivíduo com idade entre 10 e 18 anos^29^, além de lidar com as transformações corporais, começa a ser inserido em relações sociais nas quais lhe são exigidas respostas comportamentais que incluem responsabilidade e autonomia^19^ ^-^ ^20^ ^,^ ^23^, diferentemente daquelas permitidas às crianças^16^ ^,^ ^19^ ^-^ ^20^ ^,^ ^22^. Portanto, buscar conhecimento sobre problemas que afetam esse grupo social contribui para práticas voltadas à saúde que atendam às suas demandas.

Os resultados mostram que a perspectiva dos familiares foi a mais investigada nos estudos em tela. É comum, na pesquisa qualitativa, a elucidação de fenômenos sob a óptica de familiares, especialmente quando se trata de questões de saúde de grupos socialmente vulneráveis, entre os quais estão incluídas, além de crianças e adolescentes, pessoas idosas e pessoas com deficiências^10^ ^,^ ^102^ ^-^ ^104^. 

Tal fato pode estar relacionado à situação de dependência desses grupos e à convivência entre o indivíduo e a família, o que aumenta a frequência das interações e favorece a manutenção das relações^2^. Portanto, infere-se que esse é o grupo social capaz de elucidar fenômenos relacionados à saúde destes indivíduos.

Blumer foi o teórico mais citado nos estudos selecionados. Pode-se inferir que isso se deve à sua importância para essa abordagem teórica, considerando sua participação na compilação dos pressupostos, conceitos e ideias propostos por seu antecessor, Mead^1^ ^-^ ^2^ ^,^ ^7^. Inclusive, Blumer foi responsável por organizar e sistematizar o IS, conforme descrito em “*Symbolic Interactionism: perspective and method*”^2^ ^,^ ^7^, a obra mais citada nos estudos selecionados.

Há de se recorrer à aplicação do referencial teórico, uma vez que este é “o *core* do pensamento do pesquisador”^105^. Devido a essa centralidade, observa-se que os autores dos estudos selecionados se empenharam em manter o pensamento teórico baseado na obra primaz do IS, cujo conteúdo foi extraído diretamente do precursor Mead^1^ ^-^ ^2^. Tal prática é recomendada^7^ ^,^ ^105^ ^-^ ^107^, por possibilitar interpretações a partir das ideias do autor original. 

A aplicação de um referencial teórico compatível com o objeto de pesquisa é prática reconhecida na construção do conhecimento científico^105^. Nos estudos selecionados, além de o IS colaborar para a visão dos pesquisadores frente aos objetos investigados, sua origem filosófica apresenta intersecção com a da TFD^3^ ^,^ ^7^. Possivelmente, esse alinhamento favorece a tessitura das obras interacionistas em estudos fundamentados na TFD^105^ ^,^ ^107^ ^-^ ^108^. 

As propostas metodológicas de Strauss guiaram a maioria dos estudos selecionados. Talvez Strauss tenha sido o estudioso que mais aprimorou os recursos metodológicos da teoria, como observado em suas parcerias com Kate Charmaz e Juliet Corbin, com quem desenvolveu a ferramenta analítica denominada “modelo paradigmático”^3^ ^,^ ^7^. 

Esse mecanismo, presente na etapa de codificação, permite o ordenamento dos dados, e foi aprimorado para contemplar três elementos: condição, ação-interação e consequência, em substituição ao que antes era conhecido como os 5Cs: contexto, condições causais, condições intervenientes, estratégias e consequências^3^ ^,^ ^109^. Segundo estudiosos, a adoção de três componentes confere maior flexibilidade aos pesquisadores^7^ ^,^ ^109^ ^-^ ^110^, o que possivelmente facilita a adoção dessa vertente^109^. 

Atualmente, são reconhecidas ao menos quatro vertentes da TFD^7^ ^,^ ^109^ ^-^ ^110^. A maioria dos textos selecionados utiliza mais de um referencial da TFD para a condução metodológica, de modo que autores discutem a necessidade de homogeneizar a aplicação do método a partir de um único referencial^108^, como evidenciam 70% dos textos selecionados.

Cumpre observar que a prescrição metodológica apresentada nos livros demanda tempo para o desenvolvimento do trabalho científico. Em busca de assegurar aprofundamento dos dados e rigor metodológico, inclui-se ao *corpus* indutivo-dedutivo da TFD a simultaneidade entre coleta e análise, com empenho em estratégias como: verificação de outras perspectivas sobre as informações; busca de subsídios teóricos em diversas fontes; consultas bibliográficas; e discussões sobre subtemas que emergem dos dados^3^ ^,^ ^7^. 

Essa análise multifatorial possibilita a construção de novas hipóteses e a formulação de perguntas que poderão ser dirigidas a outros grupos sociais que vivenciam o fenômeno investigado^3^ ^,^ ^4^ ^,^ ^110^. Essa dinâmica da TFD visa ao preenchimento de vazios temáticos, à confirmação ou refutação de hipóteses e à complementariedade de informações diante de sua evidente escassez, a fim de atribuir maior amplitude e variedade aos dados.

Tais elementos colaboram para o aprofundamento do fenômeno, orientam as reflexões do pesquisador e o levam a “girar” ao redor do fenômeno, com observações, vivências e perspectivas distintas (ou similares), que enriquecem os conceitos que o constituem^3^ ^,^ ^5^ ^,^ ^103^ ^,^ ^110^. 

Para tanto, é preciso garantir a existência de características distintas em cada grupo amostral - elemento essencial para a escolha dos próximos grupos, pois as distinções do grupo subsequente em relação ao anterior colaboram para a cobertura das lacunas identificadas nos dados. Esse processo intensivo de coleta e análise demanda experiência em estudos qualitativos e tempo do investigador científico^3^ ^-^ ^4^ ^,^ ^6^ ^,^ ^110^ ^-^ ^111^, motivo pelo qual alguns estudiosos demoraram tempo igual ou superior a 10 meses para finalizar esse processo.

A entrevista foi a única estratégia de coleta de dados na maioria dos estudos selecionados. Entretanto, na obra mais recente da TFD^3^, recomenda-se a combinação de estratégias/técnicas para a coleta de dados. Isso se justifica porque a utilização de múltiplas técnicas é favorável ao desenvolvimento desse tipo de pesquisa, pois algumas servem para a imersão do pesquisador no campo, para a abordagem de temas sensíveis ou para a aproximação com participantes da pesquisa, assim como a técnica da observação (participante ou não-participante)^3^ ^,^ ^24^ ^,^ ^108^. Adiciona-se, ainda, o uso da técnica do desenho-história, aplicada com êxito a grupos vulneráveis e temas sensíveis^3^ ^,^ ^24^ ^,^ ^110^ ^,^ ^112^ ^-^ ^114^.

Assim, o emprego de múltiplas formas de coleta de dados precisa ser compatível com as peculiaridades encontradas em cada cenário de pesquisa e em cada grupo social investigado^3^ ^,^ ^110^. Pondera-se, também, que a observação contribui para o levantamento de dados próprios da convivência dos atores sociais em seu cotidiano e que, por vezes, podem demonstrar ao pesquisador uma realidade que não foi evidenciada pela fala durante a entrevista^24^ ^,^ ^103^ ^,^ ^110^, mas que é perceptível com a convivência do pesquisador com o grupo pesquisado, especialmente quando esse pesquisador é um agente externo ao cenário de pesquisa. 

Logo, agregar diferentes técnicas de coleta favorece o desenvolvimento de teorias substantivas e formais. Desse modo, sugere-se aos pesquisadores que optam pelo método TFD, sustentado pela lente filosófica do IS, o investimento em variedade de técnicas para a coleta de dados^3^, inovação que enriquece a experimentação do pesquisador no campo de investigação. 

Segundo estudos^107^ ^-^ ^108^ ^,^ ^111^, diagramas e memorandos são usados na maioria das teses e dissertações analisadas, pois auxiliam na reflexibilidade necessária na TFD^3^ ^,^ ^5^ ^,^ ^24^ ^,^ ^106^ ^,^ ^115^ ^-^ ^116^. Contraditoriamente, esta revisão alerta para a necessidade de incremento desses recursos analíticos, pois mais da metade dos textos não mencionou seu uso. Ademais, observa-se a escassez de estudos metodológicos que elucidem os caminhos percorridos pelos autores^103^ ou que busquem lacunas científicas a partir do exame da aplicação teórica-metodológica de estudos qualitativos. 

De fato, reconhece-se a importância de descrever os percursos metodológicos em estudos complexos como a TFD^24^ ^,^ ^103^ ^,^ ^108^. Contudo, pondera-se que pesquisadores esbarram em regras para publicação que, além de desafiar o poder de síntese do autor, limitam cada vez mais o registro detalhado do percurso metodológico na escrita de artigos, haja vista a exigência de reportar de forma qualificada a conduta metodológica empregada^117^, sendo o pesquisador obrigado a apresentar, de maneira restrita, todo o caminho seguido durante a pesquisa.

Essa realidade é identificada em pesquisa que descreve o processo de amostragem teórica no desenvolvimento de uma TFD na vertente Straussiana, ao elucidar as explicações/informações usadas na pesquisa matriz que fomentaram as escolhas de grupos amostrais e, consecutivamente, culminaram na amostragem teórica^103^. Na TFD, tais escolhas precisam ser explicadas/justificadas e ter relação basal com os dados^3^ ^,^ ^7^ ^,^ ^106^ ^,^ ^110^ ^-^ ^111^.

Poucos *softwares* são utilizados no processo analítico, pois somente 7 (11,86%) textos utilizaram algum programa de computador nessa etapa. Uma revisão com 239 estudos destaca a predominância do uso de *softwares* na etapa analítica de pesquisas qualitativas em teses e dissertações. Apesar das dificuldades elencadas quanto à utilização desse recurso, os pesquisadores descrevem suas potencialidades, como a otimização do tempo de análise de grande volume de material e a possibilidade de estabelecimento de padrões analíticos^118^, condições aconselháveis em estudos de TFD.

O NVivo foi o recurso mais utilizado, embora outra pesquisa revele a existência de 11*softwares*diferentes disponíveis para pesquisas qualitativas^118^. Infere-se que a utilização do NVivo decorra da sua longevidade e popularidade entre pesquisadores, pois esse recurso encontra-se na sua 15ª versão, o que sugere maior difusão entre pesquisadores. Em suma, o NVivo facilita a preparação de dados para análise, exibição e elaboração de relatórios^3^ ^,^ ^115^. 

Esse *software* tem capacidade de importação de diversos tipos de arquivos e dá acesso a dados estatísticos, textuais, recursos visuais digitais que podem ser utilizados como dados^3^ ^,^ ^115^. Ou seja, contempla a análise de uma variedade de informações, como requerem algumas pesquisas qualitativas^115^ ^-^ ^116^, sobretudo a TFD^3^ ^,^ ^7^ ^,^ ^119^ ^-^ ^120^. Estudiosos concordam que o uso do *software* atribui agilidade à pesquisa, pois auxilia na execução da análise^116^ ^,^ ^118^ ^,^ ^120^, além de colaborar para o rigor metodológico ao aproximar os dados coletados das idas e vindas ao campo, característica da TFD^3^ ^,^ ^119^. 

Neste período o pesquisador fica imerso nos dados, sendo essencial condensar grande número de informações^3^ ^,^ ^107^ ^,^ ^115^. As fases de codificação, agrupamento e surgimento de categorias e subcategorias tornam esse processo analítico intenso^109^ ^,^ ^118^, cuja organização e armazenamento dos dados são favorecidos pelo *software* ^3^ ^,^ ^115^ ^-^ ^116^ ^,^ ^120^, o que é benéfico e seguro ao pesquisador, que precisa honrar a fidelidade aos dados brutos.

Estudo alerta para a escassez de TFDs cuja validação da teoria não é apresentada no método^107^, assim como ocorre nos estudos selecionados. Embora não tenha sido sinalizada em todos os estudos selecionados, a validação da teoria emergente é essencial para a verificação de sua aplicabilidade, compreensão, generalização e controle, características que permitem amplitude, profundidade e variação das dimensões constatadas nos dados e embasadas na realidade^3^ ^,^ ^4^ ^,^ ^110^. 

Ademais, pesquisadores reconhecem a necessidade de validação da teoria, inclusive em outras realidades sociais^3^ ^,^ ^7^ ^,^ ^110^. Por isso, em respeito ao método, estudiosos validaram a teoria em discussões com especialistas no método e com expertises no assunto investigado^113^ ^,^ ^121^ ^-^ ^125^, enquanto outros agregaram outra abordagem metodológica para a validação^119^ ^,^ ^126^ ^-^ ^127^ ou confirmaram o fenômeno em entrevistas com adolescentes suecos^128^. Destarte, tal qual sugere a flexibilidade proposta pelo método^3^ ^,^ ^110^, as estratégias de coleta, análise e validação podem e devem ser adequadas às possibilidades do investigador e às condições encontradas no campo de pesquisa.

Por fim, o contexto mais investigado foi o serviço de saúde hospitalar, seguido do especializado. Talvez porque esses são ambientes controlados, nos quais o pesquisador consegue observar com facilidade a reunião de pessoas que implicitamente vivenciam um mesmo fenômeno. Por outro lado, ambientes de atendimento especializado em situações emergenciais de saúde constituem estruturas de acesso restrito que impõem obstáculos à coleta de dados, especialmente por meio de entrevistas em profundidade, situação vivenciada em estudos qualitativos realizados em hospitais do Nordeste brasileiro^13^ ^,^ ^102^.

Há evidente ausência de pesquisas realizadas em países da África, América Central e na Antártica, enquanto, no cenário da América do Sul, destacaram-se estudos brasileiros. Coincidentemente com outra revisão^114^, as pesquisas de TFD na área da saúde da criança e do adolescente concentraram-se no Sudeste e no Sul, regiões que reúnem maior número de universidades e centros de investigação científica no Brasil.

Este estudo tem como limitação não alcançar o mapeamento de pesquisas compostas por crianças e adolescentes como grupos amostrais separados, visto que não apresentam resultados com distinção de faixa etária nessas populações, apesar da especificidade de cada fase do desenvolvimento. Não obstante, resultou em uma varredura relevante na identificação de lacunas científicas capazes de motivar futuras produções sobre temas latentes. 

Aponta também para a possibilidade de novas revisões qualitativas sobre temas de interesse do campo social e da saúde. Ao mesmo tempo, sugere a necessidade de maior participação de crianças e profissionais em futuras pesquisas para cobrir as lacunas aqui postas.

Recomenda-se observar temáticas que preencham os vazios concernentes à aplicação teórico-metodológica proposta, a fim de suscitar novos e inéditos trabalhos de pesquisa científica à luz do IS sob o delineamento metodológico da TFD. Por fim, é aconselhável a realização de outras revisões semelhantes que abarquem a literatura cinzenta (especialmente teses e dissertações), para fins de comparação com os resultados alcançados.

## Conclusão

Adolescentes e familiares representam a maioria dos participantes dos estudos selecionados. Destaca-se a autoria de Blumer e Strauss, respectivamente, como referencial teórico e metodológico, sendo que a maioria dos estudos utilizou apenas a entrevista como estratégia para coleta de dados. Os pesquisadores dos estudos selecionados recorrem aos recursos analíticos da TFD, como diagramas, memorandos e *softwares*, de forma restrita, bem como à validação da teoria substantiva. O serviço hospitalar e o especializado foram os contextos mais pesquisados, com destaque para a maioria de estudos realizados no Brasil. 

A escassez de estudos realizados com a colaboração simultânea de crianças, adolescentes, familiares e profissionais constitui uma lacuna apontada neste estudo. Ainda, o presente estudo serve de incentivo à utilização de estratégias/técnicas de coleta de dados combinadas que possibilitem a imersão do pesquisador no campo, a aproximação com o grupo investigado e que colaborem para maior expressão da realidade por meio dos dados.

Alerta-se para a necessidade de utilização de recursos analíticos que contribuam para o rigor metodológico, um dos vazios apontados por esta revisão. Além disso, recomenda-se a realização de estudos em estados e países com contextos socioculturais diferentes, a fim de fomentar a comparação constante. 

Sugere-se ampliar o espectro das investigações científicas na área da saúde da criança e do adolescente com referencial teórico-metodológico similar, de maneira a possibilitar a busca de informações com profissionais de forma interdisciplinar e/ou interprofissional, com vistas ao desenvolvimento e aprofundamento de informações que contribuam para a construção de teorias substantivas.

## Data Availability

O conjunto de dados deste artigo está disponível sob o DOI 10.6084/m9.figshare.30675572.

# Translation: Interaccionismo Simbólico y Teoría Fundamentada en investigaciones sobre la salud de niños y adolescentes: revisión de alcance^*^

**DOI:** 10.1590/1518-8345.7900.4932

**Authors:** Marimeire Morais da Conceição, Climene Laura de Camargo, Maria Ribeiro Lacerda, Maria Carolina Ortiz Whitaker, Amâncio António de Sousa Carvalho, Kamylla Santos da Cunha, Ariane Teixeira de Santana, Telmara Menezes Couto, Eliana Rosa da Fonseca, Cristiane Cardoso de Paula

**Affiliations:** 1 Universidade Federal da Bahia, Salvador, BA, Brasil.; 2 Secretaria de Saúde da Bahia, Escola de Saúde Pública da Bahia Professor Jorge Novis, Salvador, BA, Brasil.; 3 Bolsista da Coordenação de Aperfeiçoamento de Pessoal de Nível Superior (CAPES), Brasil.; 4 Bolsista do Conselho Nacional de Desenvolvimento Científico e Tecnológico (CNPq), Brasil.; 5 Universidade Federal do Paraná, Curitiba, PR, Brasil.; 6 Universidade de Trás-os-Montes e Alto-Douro, Escola Superior de Saúde, Quinta dos Prados, Vila Real, Portugal.; 7 Centro de Investigação em Estudos da Criança, Universidade do Minho, Braga, Portugal.; 8 Universidade Federal de Santa Catarina, Florianópolis, SC, Brasil.; 9 Governo Municipal, Secretaria Municipal de Saúde de Biguaçu, Biguaçu, SC, Brasil.; 10 Universidade Federal do Rio de Janeiro, Rio de Janeiro, RJ, Brasil.; 11 Universidade Federal da Santa Maria, Santa Maria, RS, Brasil.; 12 Instituto JBI, Centro Colaborador, São Paulo, SP, Brasil.

**Keywords:** Interaccionismo Simbólico, Teoría Fundamentada, Salud del Adolescente, Niño, Revisión, Salud.

## Abstract

**Objetivo:**

mapear la aplicación teórico-metodológica del Interaccionismo Simbólico y de la Teoría Fundamentada en investigaciones sobre la salud de niños y adolescentes.

**Método:**

revisión de alcance conducida según las recomendaciones del JBI. Se consideraron: Participantes - niños y/o adolescentes, familiares y profesionales; Conceptos - Interaccionismo Simbólico y Teoría Fundamentada en los Datos; Contexto - no delimitado. Se consultaron nueve fuentes electrónicas, incluyendo artículos originales, sin restricción idiomática o temporal. Para la selección y síntesis se utilizaron los programas Rayyan y NVivo.

**Resultados:**

se seleccionaron 59 estudios. La mayoría de los participantes fueron adolescentes (49%) y familiares (40%). Respecto a la perspectiva teórica, el 42% utilizó el referencial de Blumer y el 67% la metodología de Strauss. La combinación de estrategias de recolección de datos se observó en el 34% de los estudios; recursos analíticos, como diagramas y memorandos, fueron mencionados en el 42% y el 39%, respectivamente. Solamente el 24% validó la teoría. El contexto hospitalario fue el más investigado (27%).

**Conclusión:**

la combinación de estrategias de recolección de datos posibilita mayor variedad de análisis, e investigaciones en diferentes contextos socioculturales e intersectoriales, con un incremento de información en la construcción de teorías sustantivas. Se recomiendan estudios futuros con participación simultánea de niños, adolescentes, familiares y profesionales.

## Destacados

**(1)** Los adolescentes y los familiares son los grupos sociales más investigados.

**(2)** Las obras de Strauss son las más citadas como referente metodológico.

**(3)** La combinación de estrategias para la recolección de datos es un recurso poco utilizado.

**(4)** Recursos analíticos, como diagramas y memorandos, son poco utilizados.

**(5)** Menos de una cuarta parte de las investigaciones menciona la validación de la teoría sustantiva.

## Introducción

El Interaccionismo Simbólico (IS) es una teoría de la Sociología que surgió a finales del siglo XVII y fue impulsada en la década de 1930. En este proceso, los estudiosos buscaban explicar la relación entre las personas y la sociedad por medio de las interacciones, así como las implicaciones de estas en el psiquismo de los individuos^1^ ^-^ ^2^. El IS parte de tres presupuestos: 1) los seres humanos actúan en relación con las cosas/situaciones en función de los significados que les atribuyen; 2) el significado de estas cosas/situaciones se origina a partir de las interacciones en la sociedad; y 3) tales significados se transforman mediante las interpretaciones que la persona realiza al enfrentarlas. En medio de estos presupuestos, el IS se fundamenta en seis conceptos: sociedad, símbolo, mente, *self*, estructura e interacción social^2^.

Así, el IS introduce discusiones sobre los conceptos del ser social, cuyas bases teóricas están ancladas en las ideas de George Herbert Mead, seguido durante años por el también sociólogo Herbert Blumer, quien reunió, organizó, estructuró y registró por escrito los pensamientos de Mead^1^ ^-^ ^2^. Ese contenido era presentado en clases, reuniones y conferencias en la Universidad de Chicago, donde se formó Anselm Strauss^2^ ^-^ ^3^. 

Strauss recibió influencias del IS y, junto con otro sociólogo, Barney Glaser, desarrolló la Teoría Fundamentada en los Datos (TFD)^3^. La TFD es una metodología de investigación que sigue el pensamiento positivista. Sin embargo, Strauss incorporó formas analíticas interpretativistas que dialogan con el IS^2^ ^-^ ^3^, dando origen a las raíces teóricas de la TFD en la investigación cualitativa^3^.

En la aplicación de la TFD, el investigador busca evidenciar las estrategias desarrolladas por los individuos al interactuar con diversos objetos sociales, especialmente situaciones vividas en su contexto de vida y en la sociedad de la que forman parte^3^ ^-^ ^5^. Por tal motivo, la aplicación de la TFD es actualmente reconocida por su éxito en investigaciones orientadas a generar teorías formales y sustantivas^3^ ^-^ ^6^. En continuidad con el propósito inicial de Glaser y Strauss, el método se muestra adecuado para abordar temáticas y procesos sociales considerados complejos y multifacéticos^3^ ^,^ ^7^, como ocurre en las investigaciones del área de la salud. 

Cabe señalar que, en el presente estudio, el término salud se corresponde con el concepto acuñado por la Organización Mundial de la Salud (OMS), ampliado de manera multidimensional. Por lo tanto, abarca no solamente el ámbito biológico, conductual y socioeconómico, sino que también incorpora condiciones ambientales y relaciones políticas^8^ ^-^ ^9^. Los estudiosos consideran las interacciones esenciales en la realización de acciones en favor de la salud de niños y adolescentes^10^ ^-^ ^13^. En consecuencia, resulta relevante centrar la aplicación teórico-metodológica de la TFD asociada al IS en investigaciones científicas sobre la salud de esta población, dado que ambos marcos favorecen la comprensión de las relaciones establecidas entre los individuos en la sociedad.

Esta experiencia social se inicia dentro del útero^14^ ^-^ ^15^, es conducida durante la infancia y la adolescencia por la familia (socialización primaria) y perdura a lo largo de la vida con la participación de profesionales de la salud y de la educación (socialización secundaria), como demuestran estudios realizados en Brasil, China, los Países Bajos y los Estados Unidos de América (EE. UU.)^11^ ^-^ ^12^ ^,^ ^16^ ^-^ ^17^. En el contexto actual de reconocimiento de derechos, es cada vez más frecuente que niños y adolescentes dispongan de información sobre sí mismos y sobre su condición de salud^18^ ^-^ ^21^, escenario que permite su participación en la toma de decisiones y en el autocuidado, en observancia de la autonomía y el protagonismo social infantojuvenil^20^ ^,^ ^22^ ^-^ ^24^, elementos que se contraponen al cuidado adultocéntrico^19^ ^,^ ^21^ ^,^ ^25^. 

Este estudio señala vacíos científicos que podrán servir para justificar la realización de nuevas investigaciones sobre temáticas latentes, a partir del uso de estrategias de recolección de datos y de la aplicación de recursos analíticos orientados al desarrollo de teorías. Asimismo, constituye un compendio de la aplicación teórico-metodológica del IS y de la TFD en las investigaciones, además de incentivar el incremento de estudios en contextos socioculturales innovadores e inéditos. Finalmente, el objetivo de esta revisión fue mapear la aplicación teórico-metodológica del Interaccionismo Simbólico y de la Teoría Fundamentada en investigaciones sobre la salud de niños y adolescentes.

## Método

### Tipo de estudio 

Revisión de Alcance (RA) basada en las recomendaciones propuestas por el JBI^26^ ^-^ ^27^ ^)^ y presentada conforme el *checklist* PRISMA *Extension for Scoping Reviews* (PRISMA-ScR)^28^. El protocolo fue registrado en el *Open Science Framework* (OSF): https://doi.org/10.17605/OSF.IO/BKTMJ. 

El equipo de revisión estuvo compuesto por tres profesionales con experiencia teórico-metodológica en IS y TFD: una revisora con conocimiento en el área de la salud del niño y del adolescente, una consultora metodológica con experiencia en revisiones de alcance según el método JBI y una bibliotecaria con experiencia en estudios de revisión en el área de la salud.

### Pregunta de revisión 

Se utilizó el mnemónico PCC^27^, que representa: (P) Población - niños, adolescentes, familiares y/o profesionales; (C) Concepto - IS y TFD; y (C) Contexto - cualquier entorno. A partir de ello, la pregunta orientadora se formuló de la siguiente manera: ¿cómo se aplican, desde el punto de vista teórico-metodológico, el IS y la TFD en las investigaciones sobre la salud de niños y adolescentes? 

Se plantearon preguntas secundarias para guiar el mapeo: ¿Qué grupos sociales son abordados en los estudios sobre la salud de niños y adolescentes? ¿Qué herramientas analíticas y metodológicas se utilizan en la producción del conocimiento? 

### Acrónimo PCC

Se definieron como participantes: 1) niños y adolescentes: de dos a 18 años incompletos, siguiendo el marco de referencia de la Organización Mundial de la Salud (OMS)^29^; 2) cuidadores familiares: personas que detentan la autoridad legítima sobre el niño y/o adolescente, que tengan vínculo familiar y/o mantengan (o no) parentesco con el niño o adolescente, como padre/madre, padrastro/madrastra, abuelo(a), tío(a), hermano(a), primo(a)^29^ ^-^ ^31^; 3) cuidadores no familiares: personas que cuidan de niños y/o adolescentes, aunque no sean miembros de la familia ni profesionales de la salud, como niñeras, docentes, supervisores(as), entrenadores(as)^29^ ^,^ ^32^; 4) profesionales de la salud: individuos con formación en el área de la salud, como personal de enfermería, médico(a), nutricionista, fisioterapeuta, fonoaudiólogo(a), trabajador(a) social, psicólogo(a), terapeuta ocupacional, Agentes Comunitarios de Salud (ACS)^29^ ^-^ ^30^ ^,^ ^33^; y 5) profesionales sin formación en salud, como educadores y agentes de protección y seguridad infantojuvenil^30^. 

En cuanto al *concepto*, se incluyeron exclusivamente estudios que investigaran las formas de aplicación teórico-metodológica del IS en investigaciones conducidas mediante el método TFD. El IS sostiene que los individuos reaccionan ante los objetos sociales (objetos, discursos, acciones, personas) recurriendo a símbolos y atribuyéndoles significados^1^ ^-^ ^2^.

La TFD es una metodología de investigación científica que orienta la construcción de teorías mediante la agrupación de conceptos. Para ello, recurre a la recolección y al análisis simultáneos, rigurosamente sistematizados para evidenciar conceptos y demostrar cómo se relacionan entre sí y en torno a un fenómeno central. La teoría emergente a partir de los datos explica las experiencias de los individuos y justifica sus reacciones frente a los acontecimientos con los que se enfrentan^3^ ^,^ ^7^. 

Se admitió cualquier contexto nacional o internacional, considerando cualquier escenario de atención a la salud de niños y adolescentes. 

Se establecieron como criterios de inclusión: artículos publicados en revistas o periódicos revisados por pares, con revisión por pares ciega, provenientes de investigación de campo, estudios originales y empíricos, de cualquier enfoque, fundamentados en el IS y cuya conducción metodológica se haya realizado a partir de la TFD. Debido a la posibilidad de duplicación de datos extraídos de los métodos de los estudios seleccionados, no se incluyó literatura gris. Se excluyeron estudios que utilizaran más de un referencial teórico-metodológico. 

### Recolección de datos

La estrategia de búsqueda fue elaborada por la bibliotecaria que integró el equipo de esta revisión. A partir del acrónimo, se identificaron los términos de búsqueda en los vocabularios controlados - Descriptores en Ciencias de la Salud (DeCS), *Medical Subject Heading* (MeSH) y *Embase Subject Headings* (Emtree) - y se realizaron dos búsquedas preliminares. Tras la definición de la estrategia de búsqueda (Figura 1), esta fue adaptada a cada base de datos.

**Figura 1:** Estrategia de búsqueda aplicada a Medline/PubMed el 08 de febrero de 2023, actualizada el 10 de julio de 2024

Búsqueda	Estratégia de Búsqueda	Resultados
#1	Search: “Grounded Theory”[mh] OR Grounded Theory[tiab] Sort by: Most Recent	14,791
#2	Search: “Symbolic Interactionism”[mh] OR Symbolic Interactionism*[tiab] OR symbolic interaction[tiab] OR symbolic interaction theory[tiab] OR “Social interaction”[mh] OR Social Functioning*[tiab] OR Social Interaction*[tiab] Sort by: Most Recent	40,810
#3	Search: “Infant”[mh] OR Infant*[tiab] OR “Child, Preschool”[mh] OR Preschool*[tiab] OR “Child”[mh] OR Child*[tiab] OR Children[tiab] OR Pediatric*[tiab] OR “Adolescent[mh]” OR Adolescence[tiab] OR Adolescent*[tiab] OR Teen*[tiab] OR Teenager*[tiab] OR Youth*[tiab] OR “Child Health”[mh] Sort by: Most Recent	3,519,518
#4	Search: (#1 AND #2) AND #3 Sort by: Most Recent	84

Las búsquedas se realizaron en las siguientes fuentes: Medline (PubMed), *Scientific Electronic Library Online* (SciELO), Base de Datos de Enfermería (por medio del Portal de la Biblioteca Virtual en Salud), *Cumulative Index to Nursing and Allied Health Literature* (CINAHL), *Education Resources Information Center (ERIC), Academic Search Scopus* (EBSCO), Embase (Elsevier), *Web of Science* (*Clarivate Analytics*) y *PsycINFO*.

Inicialmente, las 1.357 referencias recuperadas fueron importadas al gestor EndNote para la identificación y eliminación de duplicados y, posteriormente, exportadas a Rayyan, programa web gratuito financiado por la *Qatar Foundation* ^34^. La selección de los textos se realizó de manera doblemente independiente, con base en los criterios de inclusión. La primera fase de cribado se efectuó mediante el análisis de título y resumen; la segunda, mediante la lectura del texto completo. En caso de desacuerdo entre las revisoras, se consultó a una tercera persona para la toma de decisión^27^. 

Las 1.947 referencias fueron analizadas tras la selección de 51 estudios, y se añadieron ocho textos adicionales. Nueve textos no localizados en versión completa fueron solicitados a los autores por correo electrónico; sin embargo, solamente uno proporcionó el texto completo, que fue incorporado a los resultados.

La revisora 1 elaboró una hoja de cálculo en Excel y una leyenda con todas las variables y sus respectivas descripciones, con el fin de uniformizar la extracción de datos^35^. Una prueba piloto^27^ permitió realizar ajustes en la hoja de cálculo. En la etapa 1 se extrajeron datos como: autores, objetivo, temática, descriptores, año de publicación y país donde se realizó la investigación.

En la etapa 2 se extrajeron los siguientes elementos: 1) participantes: muestra; participación de niños y/o adolescentes (cantidad, edad, sexo, raza/color); participación de familiares (cantidad, edad, sexo, raza/color, vínculo con el niño/adolescente); profesionales (cantidad, edad, sexo, raza/color, área de actuación); 2) conceptos: en el IS, autores citados, obras citadas y aplicación de los seis conceptos; en la TFD, autores, obra citada, enfoque metodológico, técnica/instrumento de recolección de datos, duración de las entrevistas, período de recolección de datos, memorandos, diagramas, número de códigos extraídos, cantidad de grupos muestrales, *software* utilizado, validación y tipo de validación; y 3) contexto: lugar de la investigación (servicios de salud, educación, comunidad, asociaciones), siendo los servicios de salud clasificados como hospitalarios, especializados o de atención básica.

Para la extracción de datos, se realizó una lectura minuciosa del material seleccionado por dos revisoras independientes^27^ ^,^ ^35^ ^-^ ^36^. Los datos fueron agrupados, sintetizados en Microsoft Excel y, posteriormente, analizados en el *software* NVivo versión 14. En Excel, los artículos recibieron un identificador numérico del 01 al 59, de acuerdo con el año y el orden de publicación.

La presencia de las variables que componían el PCC fue señalizada en la hoja de cálculo de forma dicotomizada, con respuestas de una sola palabra (como “sí” o “no”), que fueron sometidas a análisis cuantitativo básico^35^, generando el porcentaje relativo al total de artículos. La distribución geoespacial de los textos publicados se realizó mediante una fórmula automática de Excel, considerando la frecuencia de realización de los estudios en diferentes países. La nube de palabras fue generada a partir del agrupamiento automático de los descriptores/palabras clave en NVivo. 

Se recurrió al análisis cualitativo descriptivo básico de contenido^35^, en el cual fragmentos de los textos fueron seleccionados, recibieron el identificador numérico asignado al artículo de origen, fueron transformados en códigos y agrupados por temas derivados de los seis conceptos basales del IS^1^ ^-^ ^2^. Posteriormente, se procedió al recuento de la frecuencia de estos elementos.

## Resultados

Se seleccionaron 59 artículos, publicados entre 1998 y 2024, de los cuales 33 (55,93%) fueron publicados en revistas nacionales y 17 (28,81%) en los últimos cinco años. Los resultados se presentan en la Figura 2 mediante el diagrama de flujo *Preferred Reporting Items for Systematic reviews and Meta-Analyses* (PRISMA)^37^:

**Figura 2:** Diagrama de flujo PRISMA^37^ de selección de los estudios. Salvador, BA, Brasil, 2024

Las investigaciones se llevaron a cabo en cinco continentes: 35 (59,32%) en América del Sur; 10 (16,94%) en Europa; 7 (11,86%) en América del Norte; 2 (3,38%) en Asia; y 1 (1,69%) en Oceanía. En América del Sur, destacan 31 (52,54%) producciones brasileñas, de las cuales 29 (49,15%) se realizaron en estados de la región Sudeste, 1 (1,69%) en la región Sur, y 2 (3,38%) no informan la región, el estado o el municipio donde se llevó a cabo la investigación. La distribución geográfica de las producciones seleccionadas se presenta en la Figura 3:

**Figura 3:** Distribución geoespacial de los estudios que informaron el lugar de realización (n = 55). Salvador, BA, Brasil, 2024

En total, 58 (98,30%) artículos presentaron descriptores extraídos del DeCS o del MeSH. En los descriptores utilizados, se observa que el término “Enfermería” (y correlatos) fue empleado en 30 (50,84%) artículos; el término “Niño”, en 25 (42,37%); y la expresión “Teoría Fundamentada”, en 11 (18,64%), datos congruentes con la Figura 4:

**Figura 4:** Nube de palabras generada a partir de los descriptores/palabras clave de los artículos (n = 58). Salvador, BA, Brasil, 2024

Los autores investigaron temáticas relacionadas con: enfermedades, 17 (28,81%); relaciones sociales, 13 (22,03%) - vinculadas a las interacciones establecidas entre los individuos^2^; y procesos sociales, 9 (15,25%) - respuestas individuales o colectivas frente a objetos, situaciones y contextos sociales^2^. Además, 7 (11,86%) estudios se relacionaron con intervenciones terapéuticas; 5 (8,47%) abordaron afecciones; 3 (5,08%) trataron síndromes; 3 (5,08%), trastornos; y 2 (3,38%), discapacidades.

En la Figura 5, los textos se presentan desde la publicación más reciente hasta la más antigua.

**Figura 5:** Síntesis de la caracterización de los artículos seleccionados: autor, país, objetivo y muestra. Salvador, BA, Brasil, 2024

Nº	Autor	Objetivo	Muestra
1	Juan-Martínez, et al.^38^	Comprender las reflexiones sobre la construcción de identidades de género y las implicaciones de la violencia en adolescentes indígenas del noreste de Oaxaca.	45
2	Queiroz, et al^.(^ ^39^	Analizar los factores que intervienen en la gestión del cuidado de enfermería al niño hospitalizado con cardiopatía reumática.	19
3	Mazaherizadeh, et al.^40^	Explorar los factores subyacentes, los procesos y las consecuencias de los trastornos por juegos en adolescentes iraníes de sexo masculino, así como los factores contextuales relacionados con dichos trastornos en el marco del singular tapiz sociocultural y psicológico de Irán.	13
4	Picolo Gimenes, et al.^41^	Comprender cómo las enfermeras se representan a sí mismas experimentando el uso del Juego Terapéutico en el cuidado de niños/adolescentes con el apoyo del Dibujo-Historia.	11
5	Rafii, et al.^42^	Explorar el proceso de vida de las madres de niños con autismo.	14
6	Novoa-Villegas, et al.^43^	Analizar los significados que niños y niñas atribuyen a la configuración de la(s) masculinidad(es) expresada(s) en la literatura infantil feminista.	10
7	Giraldo-Giraldo, et al.^44^	Indagar la relación entre la apropiación y el uso de las redes sociales virtuales y la construcción del sí mismo en adolescentes escolares.	19
8	Moreira-Dias, et al.^45^	Presentar un modelo teórico sobre el contexto interaccional entre profesionales de la salud y familias de niños y adolescentes en cuidados paliativos.	10
9	Gimenes, et al.^46^	Comprender qué significados atribuyen los enfermeros a su rol en la interacción con niños al utilizar Juegos Terapéuticos y diseñar un Modelo Teórico representativo de esa experiencia.	11
10	Sethi^47^	Ampliar el conocimiento sobre las influencias en la crianza de los hijos desde una perspectiva ocupacional.	12
11	Pérez-Rivera, et al.^48^	Presentar y discutir los significados y las prácticas relacionadas con la paternidad y la masculinidad en la crianza de los hijos, que conducen a la reproducción social de patrones hegemónicos patriarcales o a la emergencia de formas alternativas de ser hombre en la sociedad actual.	12
12	Riordan^49^	Presentar un método y una estructura para el análisis de la pedagogía basada en video.	42
13	Layton^50^	Comprender cómo los estudiantes construyen narrativas sobre la presencia policial en sus escuelas a partir de sus experiencias con los agentes de recursos escolares (SRO).	17
14	Johnston, et al.^51^	Desarrollar una teoría fundamentada sustantiva para explicar cómo los estudiantes experimentan las expectativas de los profesores hacia ellos.	59
15	Bonelli, et al.^52^	Comprender la experiencia de las familias en el cuidado de niños con mielomeningocele.	28
16	Riordan, et al.^53^	Aclarar algunos de los tipos de problemas pedagógicos que encuentran profesores y estudiantes (presentados como una Tipología de Problemas Pedagógicos), así como comprender y explicar cómo los participantes intentaron resolver dichos problemas.	64
17	Vieira, et al.^54^	Comprender la experiencia de la sobrecarga materna en el cuidado de hijos con cáncer desde la perspectiva de la madre.	6
18	Forsberg^55^	Explorar cómo niños de 11 a 15 años hablan sobre sus mundos sociales y sobre incidentes sociales, como el acoso.	36
19	Thornberg, et al.^56^	Examinar, en detalle, cómo una pequeña muestra de estudiantes de educación secundaria explica por qué ocurre el acoso y desarrollar un análisis de teoría fundamentada basado en las perspectivas de los estudiantes.	17
20	Vieira, et al.^57^	Comprender la experiencia de la familia de un niño que utiliza un implante coclear (IC).	32
21	Faria, et al.^58^	Comprender el significado de la reinserción familiar atribuido por la familia que la vivencia y construir un modelo teórico representativo de dicha experiencia.	20
22	Forsberg^59^	Explorar las perspectivas de 40 niñas suecas de 7º y 8º grado sobre el acoso escolar.	40
23	Salazar-Blanco, et al.^60^	Reconocer el proceso formativo y transformador de la práctica pediátrica de docentes del Área de Niñez de la Facultad de Medicina de la Universidad de Antioquia.	19
24	Forsberg, et al.^61^	Explorar cómo y de qué manera los niños discuten el acoso, con el fin de ampliar el conocimiento sobre qué procesos son importantes para ellos.	48
25	Gomes, et al.^62^	Comprender los significados atribuidos por familiares cuidadores del niño en el entorno hospitalario acerca de sus interacciones con los profesionales del equipo de enfermería.	15
26	Babler, et al.^63^	Obtener una mayor comprensión de las experiencias de adolescentes que viven con diabetes mellitus tipo 1 (DM1) y crear un paradigma teórico.	11
27	Shannon, et al.^64^	Explorar las perspectivas de 21 docentes de educación preescolar, ubicados en los Estados Unidos, sobre el desarrollo profesional (DP) que recibieron.	21
28	Leksell, et al.^65^	Ampliar el conocimiento sobre cómo los niños diagnosticados con artritis idiopática juvenil (AIJ) perciben su salud bucal y la atención odontológica.	15
29	Andrade, et al.^66^	Comprender el significado de cuidar a un hijo para la madre adolescente menor de edad, develar las demandas del cuidado y construir un modelo teórico sobre dicha experiencia.	9
30	Mieto, et al.^67^	Comprender cómo las madres significan la experiencia de acompañar a su hijo en una Unidad de Hemodiálisis Pediátrica y construir un modelo teórico representativo de esa experiencia.	11
31	Dupas, et al.^68^	Comprender la experiencia del padre de un niño con cáncer, identificando qué cambios significativos provocó la enfermedad en su forma de concebir la paternidad.	5
32	Vieira, et al.^69^	Retratar la experiencia familiar ante el descubrimiento de la discapacidad auditiva de un hijo.	32
33	Mendes, et al.^70^	Comprender cómo los enfermeros de pediatría perciben el proceso de formación de alianza con los padres en el ámbito de la salud e identificar las acciones que desarrollan en ese contexto.	12
34	Mendes, et al.^71^	Describir la experiencia de las madres, el significado y la importancia que atribuyen a su participación en el proceso de cuidado de sus hijos, así como identificar las intervenciones en las que estuvieron involucradas.	18
35	Nunes, et al.^72^	Desarrollar un modelo teórico sobre la experiencia de familias de niños con síndrome de Down en edad preescolar y escolar.	21
36	Nunes, et al.^73^	Comprender cómo es para la familia tener un niño con síndrome de Down entre sus miembros.	21
37	Souza, et al.^74^	Comprender el significado de tener anemia falciforme para niños de 3 a 12 años de edad.	8
38	Haraldsson, et al.^75^	Generar un modelo teórico de los procesos sociales subyacentes que desencadenan el estrés en la vida cotidiana de las adolescentes.	14
39	Damião, et al.^76^	Comprender cómo el adolescente con diabetes mellitus tipo 1 vive su experiencia de enfermedad y cómo afronta esta situación en su vida cotidiana.	10
40	Vargens, et al.^77^	Analizar la decisión de la adolescente respecto al embarazo, con base en el significado que ella atribuye al fenómeno.	16
41	Soares, et al.^78^	Describir el fenómeno de la maternidad en usuarias de un ambulatorio de salud mental.	10
42	Vieira, et al.^79^	Comprender la vivencia del niño con insuficiencia renal crónica y analizar el significado que atribuye a dicha experiencia.	8
43	Mendes, et al.^80^	Comprender la dinámica familiar en la experiencia del trasplante hepático pediátrico e identificar las demandas y recursos de la familia.	9
44	Sano, et al.^81^	Investigar las percepciones de las madres sobre la participación de padres no residentes en familias de bajos ingresos.	83
45	Pinto, et al.^82^	Comprender cómo el adolescente con diabetes mellitus tipo 1 vive su experiencia de enfermedad.	8
46	Rossato, et al.^83^	Comprender el significado de la experiencia de la familia de un niño con dolor derivado de artritis reumatoide juvenil y construir un modelo teórico representativo de dicha experiencia.	27
47	Nunes, et al.^84^	Identificar los significados que la situación de enfermedad crónica (diabetes) genera en la dinámica familiar.	29
48	Moreira, et al.^85^	Comprender la experiencia del niño en su vivencia con la enfermedad.	12
49	Pinto, et al.^86^	Comprender el significado de las interacciones vividas por la familia durante la hospitalización de un niño de su núcleo, identificar las demandas familiares y construir un modelo teórico representativo de dicha experiencia.	7
50	Damião, et al.^87^	Comprender la experiencia del adolescente al vivir con una enfermedad crónica como la diabetes mellitus tipo 1.	7
51	Castro, et al.^88^	Comprender el significado de las interacciones vividas en el período postoperatorio de postectomía e identificar estrategias durante la experiencia quirúrgica.	12
52	Nunes, et al.^89^	Comprender cómo es para la madre tener un hijo diabético y cómo se configura su relación con el niño, la enfermedad y el tratamiento.	6
53	Moreira, et al.^90^	Comprender el significado que el niño, en el rango etario de 7 a 12 años, atribuye a la salud y a la enfermedad.	27
54	Pauli, et al.^91^	Conocer las creencias de las enfermeras en relación con la atención humanizada en la Unidad de Cuidados Intensivos Pediátricos (UCIP).	5
55	Zem-Mascarenhas, et al.^92^	Conocer la experiencia del niño institucionalizado.	30
56	Bousso, et al.^93^	Comprender el funcionamiento de la dinámica familiar del niño hospitalizado en una Unidad de Cuidados Intensivos Pediátricos (UCIP) y construir un modelo teórico sobre la experiencia de la familia que vivencia la hospitalización del niño en la UCIP.	24
57	Damião, et al.^94^	Comprender cómo la familia vivencia los períodos de dificultad impuestos por la enfermedad crónica del niño.	8
58	Oliveira, et al.^95^	Comprender las interacciones vividas, identificar los significados que el familiar acompañante atribuye a la experiencia de la hospitalización del niño, identificar lo que dicha vivencia provoca en la vida del familiar y construir un modelo teórico representativo de la experiencia.	18
59	Woodgate^96^	Obtener descripciones detalladas de las experiencias de enfermedades crónicas en adolescentes.	23
Total			1166

En 24 (40,67%) estudios, los familiares fueron objeto de investigación científica, conformando un grupo de 416 participantes, con predominio de madres en 22 (37,28%) producciones. Solamente 12 (20,33%) investigaron profesionales, siendo 7 (11,86%) con predominio del área de la Salud, 6 (10,16%) sobre profesionales de Enfermería y 5 (8,47%) del área de la Educación.La participación de adolescentes se observó en 29 (49,15%) estudios. De estos, 24 (40,67%) especificaron la edad de los adolescentes participantes. De los 27 (45,76%) artículos que especificaron el género de los adolescentes participantes, 25 (42,37%) investigaron niñas. Solamente en 15 (25,42%) textos participaron niños, totalizando 209 participantes con edades entre 3 y 9 años, con participación de niños en 15 (25,42%) y de niñas en 12 (20,33%) estudios. 

En la aplicación del concepto “Interaccionismo Simbólico”, el teórico más estudiado fue Blumer, mencionado en 25 (42,37%) artículos, seguido de Mead (22 artículos). La obra más citada fue “*Symbolic Interactionism: perspective and method*”, mencionada en 25 (42,37%) artículos. Los seis conceptos del IS fueron aplicados en los resultados, discusiones y consideraciones finales de los estudios, predominando interacción social en 52 (88,13%), sociedad en 41 (69,49%), símbolo en 39 (66,10%), *self* en 27 (45,76%), estructura en 19 (32,20%) y mente en 7 (11,86%) textos.

En cuanto al concepto “Teoría Fundamentada en los Datos”, 57 (96,61%) textos citaron al menos un material científico como referencia. El autor más citado fue Strauss en 40 (67,79%) textos, seguido de Glaser en 27 (45,76%), Corbin en 21 (35,59%) y Charmaz en 12 (20,33%). En los 53 (89,83%) artículos que utilizaron libros en la conducción metodológica, se evidenció que “*The Discovery of grounded theory: Strategies for qualitative research*” fue la obra que más orientó a los autores, presente en 37,28% de los textos. 

En cuanto al enfoque, 58 (98,30%) fueron estudios cualitativos y solamente 1 (1,69%) fue cuantitativo-cualitativo. Predominaron como instrumentos de recolección de datos: 44 (74,57%) guías de entrevistas semiestructuradas; 6 (10,16%) entrevistas abiertas/en profundidad; 2 (3,37%) juguetes terapéuticos; y 2 (3,37%) grabaciones en video. En 39 (66,10%) artículos, la entrevista fue la única estrategia de recolección de datos. En los demás textos, además de la entrevista, se utilizaron dos o más estrategias/técnicas: 5 (8,47%) entrevistas grupales/grupos focales; 1 (1,69%) dibujo-historia; y 1 (1,69%) intervención. Asimismo, 15 (25,42%) estudios incorporaron a la entrevista la técnica de observación/observación participante. 

Solamente 28 (47,45%) textos informaron el período de recolección de datos, que varió entre dos y 36 meses; en 17 (28,81%) estudios, el tiempo de recolección fue igual o superior a 10 meses. En cuanto a la duración de las entrevistas, solamente 34 (57,62%) textos registraron este dato. Específicamente, 14 (23,72%) estudios mencionaron una duración mínima de 10 minutos y máxima de 100 minutos; algunos especificaron exactamente el tiempo total de grabación, con un mínimo de 388 minutos y un máximo de 990 minutos. 

Además, solamente 27 (45,76%) estudios mencionaron la formación de Grupos Muestrales (GM). Entre los artículos, 18 (30,50%) presentaron hasta tres GM; 7 (11,86%) de cuatro a seis grupos muestrales; y 2 (3,38%) más de siete GM. Únicamente 14 (23,72%) estudios registraron las justificaciones para el cambio de GM, y solamente 3 (5,08%) mencionaron el número de códigos utilizados [12.001^48^, 21.436^43^ e 21.612^53^].

Como estrategia analítica, 23 (38,98%) estudios mencionaron el uso de memorandos/memos, y 25 (42,37%) presentaron diagramas. Solamente 7 (11,86%) artículos utilizaron *software* para el análisis, y 3 (5,08%) emplearon NVivo. De manera similar, la validación de la teoría se realizó en 14 (23,72%) estudios: 5 (8,47%) mediante presentación del modelo teórico; 2 (3,38%) entrevistas; 2 (3,38%) conversaciones; 2 (3,38%) guías/protocolos; y 1 (1,69%) grupo focal.

Del total de 27 (45,76%) investigaciones realizadas en servicios de salud, la Atención Hospitalaria y la Atención Especializada fueron campos de recolección de datos en 16 (27,11%) y 11 (18,64%) estudios, respectivamente. De los 29 (48,27%) estudios realizados en otros contextos, 16 (27,11%) se desarrollaron en escuelas; 2 (3,38%) en programas sociales; 1 (1,69%) en un grupo de apoyo; 1 (1,69%) en una institución religiosa; y 1 (1,69%) en una asociación.

## Discusión

Este estudio evidenció que los artículos de investigación que utilizan la TFD y el IS tienen como población principalmente a adolescentes y familiares. Se destaca la aplicación teórico-metodológica de las propuestas de Blumer y Strauss, respectivamente, como referentes teórico y metodológico. Se observa el predominio del uso de la entrevista como única estrategia de recolección de datos, así como un uso limitado de recursos analíticos, tales como memorandos, diagramas y *softwares*. La validación de la teoría se realizó en pocos estudios. Las investigaciones se desarrollaron mayoritariamente en el Sudeste brasileño y, en menor proporción, en contextos hospitalarios y especializados de la salud.

Los estudios seleccionados abordan intervenciones terapéuticas, afecciones, enfermedades y trastornos. Esto concuerda con los intereses sociales, ya que la salud del niño y del adolescente constituye un campo de investigación que fundamenta diversas políticas públicas nacionales e internacionales. Además, los Objetivos de Desarrollo Sostenible (ODS) inciden en estas políticas, orientando necesidades infantojuveniles con base en evidencias científicas^19^ ^,^ ^29^ ^-^ ^30^ ^,^ ^97^ ^-^ ^99^.

Esta realidad deriva de los impactos que afectan a niños y adolescentes como consecuencia de afecciones, enfermedades y trastornos. Tales condiciones, por medio de las interacciones, repercuten individualmente en sus cuidadores; sin embargo, también se observan efectos en el escenario general de salud, social y financiero de un país, así como ante la comunidad internacional^13^ ^,^ ^98^ ^-^ ^100^. 

Buscar soluciones y garantizar la resolución de estos problemas constituye un desafío para la ciencia, con la cual las investigaciones asumen un compromiso social^101^, razón por la cual “Niño” fue el descriptor más utilizado en los estudios. No obstante, y de manera contradictoria, los adolescentes fueron el grupo social más entrevistado en la muestra seleccionada.

La adolescencia se considera una etapa compleja, ya que el individuo entre 10 y 18 años^29^, además de enfrentar transformaciones corporales, comienza a integrarse en relaciones sociales en las que se le exigen respuestas conductuales que incluyen responsabilidad y autonomía^19^ ^-^ ^20^ ^,^ ^23^, a diferencia de aquellas permitidas a los niños^16^ ^,^ ^19^ ^-^ ^20^ ^,^ ^22^. Por lo tanto, generar conocimiento sobre los problemas que afectan a este grupo social contribuye al desarrollo de prácticas de salud orientadas a atender sus demandas.

Los resultados muestran que la perspectiva de los familiares fue la más investigada en los estudios analizados. En la investigación cualitativa es común la elucidación de fenómenos desde la óptica de los familiares, especialmente cuando se trata de cuestiones de salud en grupos socialmente vulnerables, entre los que se incluyen, además de niños y adolescentes, personas mayores y personas con discapacidad^10^ ^,^ ^102^ ^-^ ^104^. 

Este hecho puede estar relacionado con la situación de dependencia de estos grupos y con la convivencia entre el individuo y la familia, lo que incrementa la frecuencia de las interacciones y favorece el mantenimiento de las relaciones^2^. Por lo tanto, se infiere que este es el grupo social capaz de esclarecer fenómenos relacionados con la salud de estos individuos.

Blumer fue el teórico más citado en los estudios seleccionados. Puede inferirse que ello se debe a su relevancia para este enfoque teórico, considerando su participación en la compilación de los presupuestos, conceptos e ideas propuestos por su predecesor, Mead^1^ ^-^ ^2^ ^,^ ^7^. Asimismo, Blumer fue responsable de organizar y sistematizar el IS, tal como se describe en “*Symbolic Interactionism: perspective and method*”^2^ ^,^ ^7^, la obra más citada en los estudios seleccionados.

Es necesario recurrir a la aplicación del referencial teórico, ya que este constituye “el núcleo del pensamiento del investigador”^105^. Debido a esta centralidad, se observa que los autores de los estudios seleccionados procuraron mantener el pensamiento teórico basado en la obra fundacional del IS, cuyo contenido fue extraído directamente del precursor Mead^1^ ^-^ ^2^. Esta práctica es recomendada^7^ ^,^ ^105^ ^-^ ^107^, pues posibilita interpretaciones a partir de las ideas del autor original. 

La aplicación de un referencial teórico compatible con el objeto de investigación es una práctica reconocida en la construcción del conocimiento científico^105^. En los estudios seleccionados, además de que el IS contribuye a la perspectiva de los investigadores frente a los objetos investigados, su origen filosófico presenta intersecciones con el de la TFD^3^ ^,^ ^7^. Posiblemente, esta convergencia favorece la integración de las obras interaccionistas en estudios fundamentados en la TFD^105^ ^,^ ^107^ ^-^ ^108^. 

Las propuestas metodológicas de Strauss guiaron la mayoría de los estudios seleccionados. Es posible que Strauss haya sido el estudioso que más perfeccionó los recursos metodológicos de la teoría, como se observa en sus colaboraciones con Kate Charmaz y Juliet Corbin, con quienes desarrolló la herramienta analítica denominada “modelo paradigmático”^3^ ^,^ ^7^. 

Este mecanismo, presente en la etapa de codificación, permite la organización de los datos y fue perfeccionado para contemplar tres elementos: condición, acción-interacción y consecuencia, en sustitución de lo que anteriormente se conocía como los 5Cs: contexto, condiciones causales, condiciones intervinientes, estrategias y consecuencias^3^ ^,^ ^109^. Según diversos estudiosos, la adopción de tres componentes otorga mayor flexibilidad a los investigadores^7^ ^,^ ^109^ ^-^ ^110^, lo que posiblemente facilita la adopción de esta vertiente^109^. 

Actualmente, se reconocen al menos cuatro vertientes de la TFD^7^ ^,^ ^109^ ^-^ ^110^. La mayoría de los textos seleccionados utiliza más de un referencial de la TFD para la conducción metodológica, de modo que los autores discuten la necesidad de homogeneizar la aplicación del método a partir de un único referencial^108^, como lo evidencian el 70% de los textos seleccionados.

Cabe señalar que la prescripción metodológica presentada en los libros demanda tiempo para el desarrollo del trabajo científico. Con el fin de asegurar la profundización de los datos y el rigor metodológico, se incorpora al corpus inductivo-deductivo de la TFD la simultaneidad entre recolección y análisis, con énfasis en estrategias como: verificación de otras perspectivas sobre la información; búsqueda de fundamentos teóricos en diversas fuentes; consultas bibliográficas; y discusiones sobre subtemas que emergen de los datos^3^ ^,^ ^7^. 

Este análisis multifactorial posibilita la construcción de nuevas hipótesis y la formulación de preguntas que podrán dirigirse a otros grupos sociales que vivencian el fenómeno investigado^3^ ^,^ ^4^ ^,^ ^110^. Esta dinámica de la TFD tiene como finalidad llenar vacíos temáticos, confirmar o refutar hipótesis y complementar información ante su evidente escasez, con el propósito de otorgar mayor amplitud y variedad a los datos.

Tales elementos contribuyen a la profundización del fenómeno, orientan las reflexiones del investigador y lo llevan a “girar” en torno al fenómeno, con observaciones, vivencias y perspectivas distintas (o similares) que enriquecen los conceptos que lo constituyen^3^ ^,^ ^5^ ^,^ ^103^ ^,^ ^110^. 

Para ello, es necesario garantizar la existencia de características distintas en cada grupo muestral - elemento esencial para la elección de los grupos siguientes, ya que las diferencias del grupo subsecuente en relación con el anterior contribuyen a cubrir las lagunas identificadas en los datos. Este proceso intensivo de recolección y análisis requiere experiencia en estudios cualitativos y tiempo por parte del investigador^3^ ^-^ ^4^ ^,^ ^6^ ^,^ ^110^ ^-^ ^111^, razón por la cual algunos estudiosos emplearon un período igual o superior a 10 meses para finalizar dicho proceso.

La entrevista fue la única estrategia de recolección de datos en la mayoría de los estudios seleccionados. Sin embargo, en la obra más reciente sobre la TFD^3^, se recomienda la combinación de estrategias/técnicas para la recolección de datos. Esto se justifica porque la utilización de múltiples técnicas favorece el desarrollo de este tipo de investigación, ya que algunas contribuyen a la inmersión del investigador en el campo, al abordaje de temas sensibles o al acercamiento con los participantes, como ocurre con la técnica de observación (participante o no participante)^3^ ^,^ ^24^ ^,^ ^108^. Asimismo, se suma el uso de la técnica del dibujo-historia, aplicada con éxito en grupos vulnerables y en temáticas sensibles^3^ ^,^ ^24^ ^,^ ^110^ ^,^ ^112^ ^-^ ^114^.

De este modo, el empleo de múltiples formas de recolección de datos debe ser compatible con las particularidades encontradas en cada escenario de investigación y en cada grupo social investigado^3^ ^,^ ^110^. Además, se considera que la observación contribuye a la obtención de datos propios de la convivencia cotidiana de los actores sociales y que, en ocasiones, pueden revelar al investigador una realidad que no fue explicitada mediante el discurso durante la entrevista^24^ ^,^ ^103^ ^,^ ^110^, pero que se hace perceptible por medio de la interacción del investigador con el grupo estudiado, especialmente cuando este es un agente externo al escenario de investigación. 

Por lo tanto, integrar diferentes técnicas de recolección favorece el desarrollo de teorías sustantivas y formales. En este sentido, se sugiere a los investigadores que optan por el método TFD, sustentado en la lente filosófica del IS, invertir en una variedad de técnicas de recolección de datos^3^, innovación que enriquece la experiencia del investigador en el campo de estudio. 

Según diversos estudios^107^ ^-^ ^108^ ^,^ ^111^, los diagramas y memorandos son utilizados en la mayoría de las tesis y disertaciones analizadas, ya que contribuyen a la reflexividad necesaria en la TFD^3^ ^,^ ^5^ ^,^ ^24^ ^,^ ^106^ ^,^ ^115^ ^-^ ^116^. No obstante, de manera contradictoria, esta revisión señala la necesidad de incrementar el uso de estos recursos analíticos, dado que más de la mitad de los textos no mencionó su utilización. Además, se observa la escasez de estudios metodológicos que esclarezcan los caminos recorridos por los autores^103^ o que busquen vacíos científicos a partir del examen de la aplicación teórico-metodológica de estudios cualitativos. 

De hecho, se reconoce la importancia de describir los recorridos metodológicos en estudios complejos como la TFD^24^ ^,^ ^103^ ^,^ ^108^. Sin embargo, se considera que los investigadores se enfrentan a normas de publicación que, además de desafiar su capacidad de síntesis, limitan cada vez más el registro detallado del recorrido metodológico en la redacción de artículos, dado el requisito de reportar de manera calificada la conducta metodológica empleada^117^, lo que obliga al investigador a presentar, de forma restringida, todo el proceso seguido durante la investigación.

Esta realidad se identifica en un estudio que describe el proceso de muestreo teórico en el desarrollo de una TFD en la vertiente Straussiana, al esclarecer las explicaciones/informaciones utilizadas en la investigación matriz que fundamentaron la elección de los grupos muestrales y, consecuentemente, culminaron en el muestreo teórico^103^. En la TFD, tales elecciones deben ser explicadas/justificadas, manteniendo una relación basal con los datos^3^ ^,^ ^7^ ^,^ ^106^ ^,^ ^110^ ^-^ ^111^.

Pocos *softwares* son utilizados en el proceso analítico, ya que solamente 7 (11,86%) textos emplearon algún programa informático en esta etapa. Una revisión con 239 estudios destaca la predominancia del uso de *software* en la etapa analítica de investigaciones cualitativas en tesis y disertaciones. A pesar de las dificultades señaladas en cuanto al uso de este recurso, los investigadores describen sus potencialidades, como la optimización del tiempo en el análisis de grandes volúmenes de material y la posibilidad de establecer patrones analíticos^118^, condiciones recomendables en estudios de TFD.

El NVivo fue el recurso más utilizado, aunque otra investigación señala la existencia de 11 *softwares* diferentes disponibles para investigaciones cualitativas^118^. Se infiere que la utilización de NVivo se deba a su longevidad y popularidad entre los investigadores, dado que este recurso se encuentra en su 15ª versión, lo que sugiere mayor difusión en el ámbito académico. En síntesis, NVivo facilita la preparación de los datos para el análisis, la visualización y la elaboración de informes^3^ ^,^ ^115^. 

Este *software* permite la importación de diversos tipos de archivos y el acceso a datos estadísticos, textuales y recursos visuales digitales que pueden utilizarse como datos^3^ ^,^ ^115^. Es decir, contempla el análisis de una variedad de información, como requieren algunas investigaciones cualitativas^115^ ^-^ ^116^, especialmente la TFD^3^ ^,^ ^7^ ^,^ ^119^ ^-^ ^120^. Los estudiosos coinciden en que el uso de *software* aporta agilidad a la investigación, ya que facilita la ejecución del análisis^116^ ^,^ ^118^ ^,^ ^120^, además de contribuir al rigor metodológico al aproximar los datos recolectados a las idas y venidas al campo, característica propia de la TFD^3^ ^,^ ^119^. 

Durante este período, el investigador permanece inmerso en los datos, siendo esencial condensar un gran volumen de información^3^ ^,^ ^107^ ^,^ ^115^. Las fases de codificación, agrupamiento y surgimiento de categorías y subcategorías convierten este proceso analítico en una tarea intensa^109^ ^,^ ^118^, cuya organización y almacenamiento de datos se ven favorecidos por el *software* ^3^ ^,^ ^115^ ^-^ ^116^ ^,^ ^120^, lo que resulta beneficioso y seguro para el investigador, quien debe garantizar la fidelidad a los datos brutos.

Un estudio advierte sobre la escasez de investigaciones en TFD cuya validación de la teoría no es presentada en el método^107^, situación que también se observa en los estudios seleccionados. Aunque no fue señalada en todos los estudios analizados, la validación de la teoría emergente es esencial para verificar su aplicabilidad, comprensión, generalización y control, características que permiten amplitud, profundidad y variación de las dimensiones constatadas en los datos y fundamentadas en la realidad^3^ ^,^ ^4^ ^,^ ^110^. 

Además, los investigadores reconocen la necesidad de validar la teoría, incluso en otras realidades sociales^3^ ^,^ ^7^ ^,^ ^110^. Por ello, en respeto al método, algunos estudiosos validaron la teoría mediante discusiones con especialistas en el método y con expertos en el tema investigado^113^ ^,^ ^121^ ^-^ ^125^, mientras que otros incorporaron otro enfoque metodológico para la validación^119^ ^,^ ^126^ ^-^ ^127^ o confirmaron el fenómeno a través de entrevistas con adolescentes suecos^128^. De este modo, tal como sugiere la flexibilidad propuesta por el método^3^ ^,^ ^110^, las estrategias de recolección, análisis y validación pueden y deben adecuarse a las posibilidades del investigador y a las condiciones encontradas en el campo de investigación.

Por último, el contexto más investigado fue el servicio de salud hospitalario, seguido del especializado. Probablemente porque estos son entornos controlados, en los cuales el investigador puede observar con mayor facilidad la reunión de personas que, implícitamente, vivencian un mismo fenómeno. Por otro lado, los entornos de atención especializada en situaciones de emergencia en salud constituyen estructuras de acceso restringido que imponen obstáculos a la recolección de datos, especialmente mediante entrevistas en profundidad, situación evidenciada en estudios cualitativos realizados en hospitales del Nordeste brasileño^13^ ^,^ ^102^.

Se observa una evidente ausencia de investigaciones realizadas en países de África, América Central y la Antártida, mientras que, en el escenario sudamericano, destacaron los estudios brasileños. Coincidentemente con otra revisión^114^, las investigaciones en TFD en el área de la salud del niño y del adolescente se concentraron en las regiones Sudeste y Sur, que reúnen el mayor número de universidades y centros de investigación científica en Brasil.

Este estudio presenta como limitación no haber logrado mapear investigaciones compuestas por niños y adolescentes como grupos muestrales separados, dado que no presentan resultados con distinción por rango etario en estas poblaciones, pese a la especificidad de cada etapa del desarrollo. No obstante, permitió realizar un rastreo relevante en la identificación de vacíos científicos capaces de motivar futuras producciones sobre temáticas latentes. 

Asimismo, señala la posibilidad de nuevas revisiones cualitativas sobre temas de interés en el ámbito social y de la salud. Al mismo tiempo, sugiere la necesidad de una mayor participación de niños y profesionales en futuras investigaciones, a fin de cubrir las lagunas aquí identificadas.

Se recomienda observar temáticas que llenen los vacíos concernientes a la aplicación teórico-metodológica propuesta, con el fin de suscitar nuevos e inéditos trabajos de investigación científica a la luz del IS bajo el delineamiento metodológico de la TFD. Finalmente, se aconseja la realización de otras revisiones similares que incluyan literatura gris (especialmente tesis y disertaciones), con el propósito de comparar los resultados obtenidos.

## Conclusión

Adolescentes y familiares representan la mayoría de los participantes de los estudios seleccionados. Se destaca la autoría de Blumer y Strauss, respectivamente, como referentes teórico y metodológico, siendo que la mayoría de los estudios utilizó únicamente la entrevista como estrategia de recolección de datos. Los investigadores de los estudios seleccionados recurrieron de forma limitada a los recursos analíticos de la TFD, tales como diagramas, memorandos y *software*, así como a la validación de la teoría sustantiva. El servicio hospitalario y el especializado fueron los contextos más investigados, con predominio de estudios realizados en Brasil. 

La escasez de estudios realizados con la colaboración simultánea de niños, adolescentes, familiares y profesionales constituye una laguna señalada en este estudio. Asimismo, el presente trabajo incentiva el uso de estrategias/técnicas combinadas de recolección de datos que posibiliten la inmersión del investigador en el campo, el acercamiento al grupo investigado y que contribuyan a una mayor expresión de la realidad por medio de los datos.

Se alerta sobre la necesidad de utilizar recursos analíticos que contribuyan al rigor metodológico, uno de los vacíos identificados por esta revisión. Además, se recomienda la realización de estudios en estados y países con contextos socioculturales diferentes, a fin de fomentar la comparación constante. 

Se sugiere ampliar el espectro de las investigaciones científicas en el área de la salud del niño y del adolescente con un referencial teórico-metodológico similar, de modo que se posibilite la búsqueda de información con profesionales de forma interdisciplinaria y/o interprofesional, con miras al desarrollo y profundización de información que contribuya a la construcción de teorías sustantivas.

## Data Availability

El conjunto de datos de este artículo está disponible en el enlace DOI 10.6084/m9.figshare.30675572.
